# Structure-Based Prediction of hERG-Related Cardiotoxicity: A Benchmark Study

**DOI:** 10.1021/acs.jcim.1c00744

**Published:** 2021-09-10

**Authors:** Teresa Maria Creanza, Pietro Delre, Nicola Ancona, Giovanni Lentini, Michele Saviano, Giuseppe Felice Mangiatordi

**Affiliations:** † CNR—Institute of Intelligent Industrial Technologies and Systems for Advanced Manufacturing, Via Amendola 122/o, 70126 Bari, Italy; ‡ Chemistry Department, University of Bari “Aldo Moro”, via E. Orabona, 4, I-70125 Bari, Italy; § CNR—Institute of Crystallography, Via Amendola 122/o, 70126 Bari, Italy; ∥ Department of Pharmacy—Pharmaceutical Sciences, University of Bari “Aldo Moro”, via E. Orabona, 4, I-70125 Bari, Italy

## Abstract

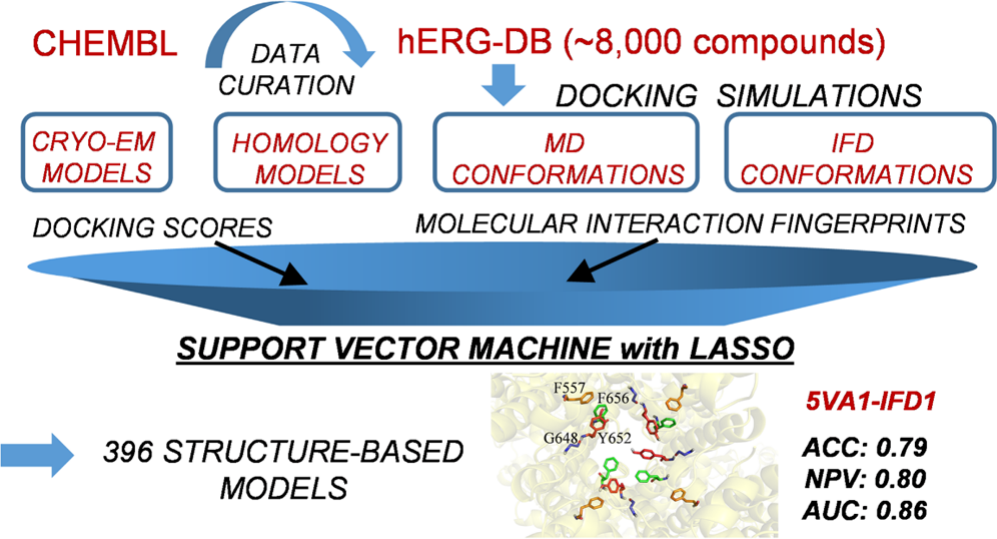

Drug-induced blockade of the human ether-à-go-go-related gene (*hERG*) channel is today considered the main cause of cardiotoxicity in postmarketing surveillance. Hence, several ligand-based approaches were developed in the last years and are currently employed in the early stages of a drug discovery process for *in silico* cardiac safety assessment of drug candidates. Herein, we present the first structure-based classifiers able to discern *hERG* binders from nonbinders. LASSO regularized support vector machines were applied to integrate docking scores and protein–ligand interaction fingerprints. A total of 396 models were trained and validated based on: (i) high-quality experimental bioactivity information returned by 8337 curated compounds extracted from ChEMBL (version 25) and (ii) structural predictor data. Molecular docking simulations were performed using GLIDE and GOLD software programs and four different *hERG* structural models, namely, the recently published structures obtained by cryoelectron microscopy (PDB codes: 5VA1 and 7CN1) and two published homology models selected for comparison. Interestingly, some classifiers return performances comparable to ligand-based models in terms of area under the ROC curve (AUC_MAX_ = 0.86 ± 0.01) and negative predictive values (NPV_MAX_ = 0.81 ± 0.01), thus putting forward the herein proposed computational workflow as a valuable tool for predicting *hERG*-related cardiotoxicity without the limitations of ligand-based models, typically affected by low interpretability and a limited applicability domain. From a methodological point of view, our study represents the first example of a successful integration of docking scores and protein–ligand interaction fingerprints (IFs) through a support vector machine (SVM) LASSO regularized strategy. Finally, the study highlights the importance of using *hERG* structural models accounting for ligand-induced fit effects and allowed us to select the best-performing protein conformation (made available in the Supporting Information, SI) to be employed for a reliable structure-based prediction of *hERG*-related cardiotoxicity.

## Introduction

Ether-à-go-go (EAG) proteins are potassium channels expressed in the muscles as well as in various brain regions, endocrine cells, and heart. The EAG-related gene (ERG) channels represent an EAG subfamily including three isoforms, namely, Kv11.1, Kv11.2, and Kv11.3, all characterized by the coassembly of four identical α-subunits each containing six transmembrane helices.^1^ Commonly known as the human ether-à-go-go-related gene (*hERG*), the human isoform Kv11.1 has attracted increasing interest over the last years since its dysfunction is associated with prolongation of the QT interval (i.e., long QT syndrome, LQTS) inducing ventricular arrhythmia (torsades de pointes, TdP), which may cause ventricular fibrillation and sudden death.^2−4^ Since LQTS can be the result not only of congenital dysfunctions but also of the drug-induced block of the channel,^5^*hERG* is today recognized as a primary antitarget in the screening of drug candidates. It is worth noting that in the last years, many pharmaceuticals from multiple drug classes including antihistamines,^6^ antiarrhythmics,^7^ antipsychotics,^8^ antimalarials,^9^ antibiotics,^10^ and gastroprokinetic^11^ were proved to induce *hERG*-related LQTS, a side effect responsible for about 30% postmarketing drug withdrawal between 1953 and 2013 in the US.^12^ In this context, a meaningful example is represented by terfenadine, an antihistamine drug removed from the market by the U.S. Food and Drug Administration (FDA) in 1997 because of its *hERG*-blocking ability.^5,13^ As a result, the assessment of *hERG*-related cardiotoxicity is today recognized as a common practice in the preclinical stages of drug discovery,^14^ in agreement with the regulatory guidelines.^15^ In this respect, different in vitro tests can be employed such as radioactive flux-based, binding, and fluorescence-based assays.^16,17^ In particular, several companies today allow screening of large collections of chemicals with a reasonable cost. In this context, *in silico* approaches are extremely appealing for their ability to support experimental toxicity testing quickly and at even lower costs.^18−20^

To this aim, several ligand-based models have been developed in the last years by employing quantitative structure–activity relationship (QSAR) approaches,^21−23^ pharmacophore models,^24−28^ and machine learning algorithms.^28−37^ The paper by Ekins et al.^24^ published in 2002 and reporting the first pharmacophore model for *hERG* inhibition is worth noting. Although developed based on few available experimental data, the model, containing one positive ionizable and four hydrophobic features, was successfully employed in the last two decades. In the same year, Cavalli et al.^26^ published a pharmacophore model showing that most of the *hERG* blockers are flexible molecules bearing a central tertiary amine function and at least two aromatic moieties.

Although ligand-based models can provide good predictive performances, their application for screening compounds spanning very different classes is limited by their restricted applicability domain^38^ as they are usually developed from training sets containing a limited number of congeneric analogues.

In this context, employing structure-based approaches, usually characterized by higher interpretability, can represent a valuable strategy to overcome this limitation^14^ and can be efficiently used in consensus strategies in combination with ligand-based classifiers.^39,40^ In particular, in the last few years, molecular docking has emerged as a valuable strategy to develop classification models in the context of predictive toxicology.^41,42^

Such a computational technique has been widely employed to shed light on the *hERG*–drug interactions, often in combination with other computational (e.g., molecular dynamics, MD)^43−45^ and experimental (mutagenesis studies) approaches,^46,47^ allowing the identification of a pool or residues responsible for drug binding in the so-called *hERG* central cavity (CC), namely, F656, Y652, G648, T623, S624, V625, and F557.^48^ As a result, although we cannot exclude the presence of other binding sites (BS) for some *hERG* binders, as postulated in some papers,^49,50^ CC is today the recognized pocket for *hERG* blockers.^51^ It is worth noting that most of these structure-based investigations were performed employing homology models based on the crystal structure of other K^+^ channels,^52−54^ as the first near-atomic resolution structure of *hERG* was determined only recently through single-particle cryoelectron microscopy. In particular, among the different models deposited by the authors,^55^ the one provided with the best resolution (3.7 Å— PDB code: 5VA1) is today emerging as the structure of choice to perform molecular docking simulations, as highlighted by the recent literature.^44,56−62^ Despite providing insights into the molecular determinants of drug binding, all of these studies focus on small data sets of compounds already proved to be (or potentially be) *hERG* binders. In other words, they do not provide any useful model for discerning *hERG* binders from safe compounds. In this paper, we present the first structure-based models for predicting the *hERG*-blocking potential of chemicals by employing a large collection of high-quality experimental bioactivity data available from ChEMBL^63^ (version 25). The models were derived by employing two popular software programs for drug discovery, namely, GLIDE^64^ v.6.5 and GOLD^65^ v.5.2 to (i) provide easy-to-run and interpretable structure-based classifiers of *hERG*-related cardiotoxicity, (ii) weigh the *hERG* structure commonly used for docking simulations as a valuable three-dimensional (3D) model for discerning safe from unsafe compounds by comparing its performance with those returned by a homology model commonly used in the last years^66,67^ and another recently proposed as able to provide docking results in agreement with experimental Ala-scan data,^44^ (iii) identify which residues are likely responsible for *hERG–*drug binding, and (iv) prompt the scientific community to consider new *hERG* structural models that, by including ligand-induced fit effects, can be used for more reliable docking simulations. From a more methodological point of view, the paper represents the first effort to develop classifiers integrating docking scores (DSs) and protein–ligand interaction fingerprints by support vector machine (SVM) LASSO regularized models, thus providing a new computational workflow for a comprehensive structure-based approach in the context of predictive toxicology.

## Materials and Methods

### Data Set Construction

A total of 17 952 activity entries were extracted from ChEMBL^63^ (version 25) according to the Target ID (ChEMBL240) assigned to the *hERG* channel. To ensure the validity of the data, the database was mined retaining only entries with the following criteria: (i) entries annotated exclusively with IC_50_ (11,144 entries) measures, (ii) data referring to assays conducted on human targets (“target_organism” = “Homo sapiens”), (iii) data marked as direct binding (“assay_type” = “B”), and (iv) entries free of warnings in the “data_validity_comment” field.^68^ In addition, molecules with molecular weights (MW) <200 or >600 Da were removed as well as duplicates. The resulting data set, hereinafter named *hERG-DB*, contains 8337 entries and is characterized by a high structural diversity as a result of the well-known *hERG* promiscuity. This is supported by the computed internal diversity (ID), namely, the average Tanimoto distance of each molecule belonging to the DB computed with respect to all of the others by employing the Morgan radius 2 fingerprint.^69^ Indeed, *hERG-DB* returned an ID value as high as 0.83.

It is worth noting that *hERG-DB* includes IC_50_ measures resulting from experiments performed on different cell lines such as HEK and CHO. However, when the purpose is that of developing classifiers rather than regression models, the noise resulting from the *hERG* IC_50_ variability can be tolerated, as confirmed by the recent literature.^28,32−34^

Consistent with previous studies,^70−73^ different inactivity thresholds (IC_50_ = 1, 10, 20, 30, 40, 50, 60, 70, and 80 μM) were used. Our training data set was therefore composed of positive and negative examples: positive molecules are those that show IC_50_ ≤ 1 μM and negative molecules are those with IC_50_ greater than the different inactivity thresholds listed above. Table S1 (see the Supporting Information) reports the number of positive and negative samples in dependence of the selected thresholds. The negative set includes also those compounds whose IC_50_ field in ChEMBL shows the expression “not a number”. As a fair comparison of classifiers requires the knowledge of distributions of the relative quality metrics,^74^ for each threshold, we trained 100 classifiers on randomly drawn negative and positive samples in the same number. This choice lets us train classifiers on balanced data sets and so prevents linear SVMs to converge on majority-class classifiers and to neglect classes of fewer samples. In particular, we performed multiple estimates of the classification performances on different external data sets: we randomly split the data into two subsets, one acting as a training set and the other as an external (validation) set, the latter including 100 compounds (50 randomly selected active and 50 randomly selected inactive compounds) unseen by the classifier. This operation was repeated 100 times by selecting each time different randomly selected external compounds. The resulting 100 performances were averaged to provide a single value of a given quality metric along with the relative standard deviation and allowed us to build a distribution used to compare the performances of the different models by statistical Kolmogorov−Smirnov (KS) tests.

### Selection and Preparation of Protein Structures

Docking simulations were performed using the following as protein structures: (i) the recently published models of the *hERG* structure, hereinafter named using their PDB IDs, namely, 5VA1^55^ and 7CN1;^75^ (ii) the homology model developed by Farid et al.^66^ using the crystal structure of the bacterial potassium channel KvAP as a template (*KvAP-Homo*); (iii) the homology model recently published by Helliwell et al.^67^ based on the X-ray crystal structure of MthK (PDB code: 1LNQ)^67^ and providing a consistent match between experimental Ala-scan and docking data returned by several *hERG* blockers (*MthK-Homo*); and (iv) two conformational states of the protein extracted from molecular dynamics (MD) simulations performed on 5*VA*1 and proposed as the protein conformations to be used to discern blockers from nonblockers (5*VA*1_*MD*_*b*) and activators from nonactivators (5*VA*1*_MD_a*) through molecular docking simulations.^44^ 5*VA*1 and 7*CN*1 were prepared using the protein preparation wizard tool^76^ available from Schrodinger Suite 2019–4,^77^ which enables us to (i) add missing hydrogen atoms, (ii) determine the optimal protonation and tautomerization states of the residues, (iii) fix the orientation of any misoriented group, and (iv) perform a final energy minimization.

### Selection of Five Representative *hERG* Binders

**Figure 1:**
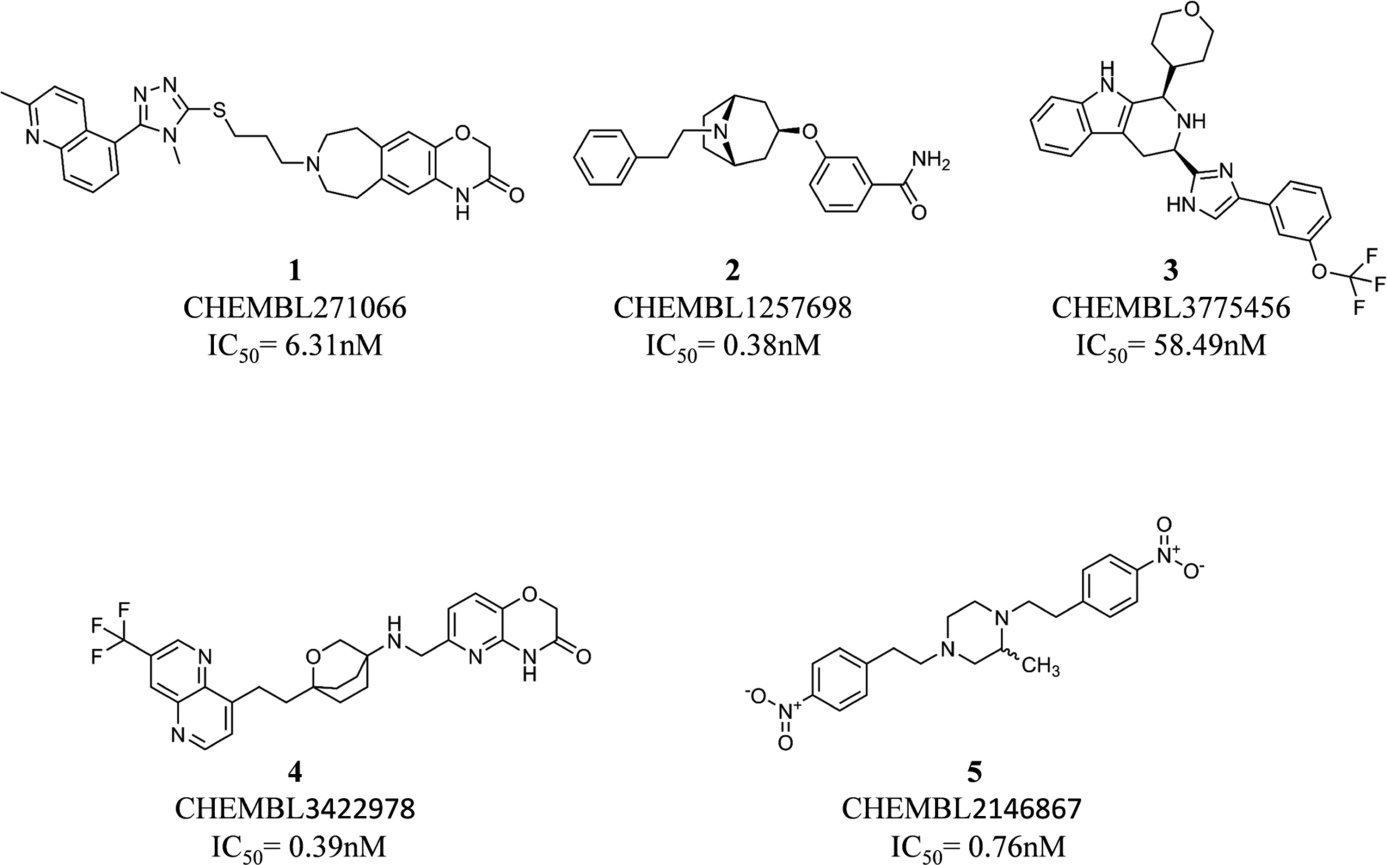
Compounds selected from the *hERG-DB* for generating *hERG* conformations using IFD simulations.

The Canvas 4.2 module^78^ of Schrödinger was used to generate binary fingerprints (i.e., MOLPRINT2D)^79,80^ of all of the compounds belonging to the *hERG-DB*. The similarity between the developed fingerprints was computed using the Tanimoto coefficient.^81^ All of the compounds were clustered into five groups using the *k*-means clustering protocol integrated into Canvas 4.2.^78^ For each cluster, the compound responsible for the lower IC_50_ value was selected for further induced-fit docking (IFD) simulations. In doing that, ligands corresponding to the following ID in ChEMBL were selected: CHEMBL271066 (IC_50_ = 6.31 nM),^82^ CHEMBL1257698 (IC_50_ = 0.38 nM),^83^ CHEMBL3775456 (IC_50_ = 58.49 nM),^84^ CHEMBL3422978 (IC_50_ = 0.39 nM),^85^ and CHEMBL2146867 (IC_50_ = 0.76 nM)^86^ (see Figure 1).

It is worth noting that the selected compounds show a molecular weight (MW) ranging from 350.46 Da (compound **2**) to 514.66 Da (compound **1**). As the majority (87.2%) of the chemicals belonging to *hERG-DB* have an MW between 300 and 550 Da, compounds **1**–**5** can be reasonably considered as representative of the whole *hERG-DB* also in terms of size.

### Induced-Fit Docking Simulations

All of the five selected compounds (Figure 1) were subjected to IFD simulations performed^87^ on 5*VA*1.^55^ All of the compounds were subjected to LigPrep^88^ to properly generate all of the tautomers and ionization states at a pH value equal to 7.0 ± 2.0. In the initial docking step, the residues known to be important for binding of *hERG* blockers, namely, F557,^67,89^ T623,^90,91^ S624,^90^ V625,^92^ Y652,^91,93^ F656,^47,93^ and G648,^47^ were mutated to alanine and the van der Waals radii of protein atoms were scaled down to 70%. A cubic grid having an edge of 10 Å for the inner box and 30 Å for the outer box centered on the residues F557, T623, S624, V625, Y652, F656, and G648 was employed. Initial docking was performed using the Glide standard precision^64^ (SP) mode and 20 poses were generated for each ligand. In the second stage, residues mutated in the initial docking step were restored and the structures of the residues within 5.0 Å of the docked ligand were refined via the Refinement module of Prime,^94^ a tool available in the Schrodinger Suite 2019-4. In the final redocking step, each ligand was docked again to the refined protein using the extra precision (XP) protocol.^64^ Finally, the generated poses were ranked using the IFD score, and the resulting top-scored protein–ligand complexes were used for further standard docking simulations.

### Standard Docking Simulations

All of the compounds belonging to the *hERG-DB* were subjected to LigPrep^88^ to properly generate all of the tautomers and ionization states at a pH value equal to 7.0 ± 2.0. Different stereoisomers were also produced in the case of entries whose chiral configuration was not defined in the *hERG-DB*. All of the selected protein structures were employed for docking simulations performed using two software programs widely used in the context of drug discovery, namely, GLIDE^64^ v.6.5, which is part of the Schrodinger Suite, and GOLD^65^ v.5.2, available as Cambridge Crystallographic Data Centre (CCDC) product. During the docking process, the receptor protein was held fixed, whereas full conformational flexibility was allowed for the ligands. The default Force Field OPLS_2005^95^ and all of the default settings for the standard precision^64^ (SP) protocol were used during docking simulations performed with GLIDE, while the scoring function CHEMSCORE^96^ was employed for docking simulations performed with GOLD. Finally, a cubic grid having an edge of 30 Å for the outer box and 10 Å for the inner box (GLIDE)^64^ and a spherical grid having a radius of 10 Å (GOLD)^65^ were centered on the center of mass of the residues F557, T623, S624, V625, Y652, F656, and G648.

It is worth noting that the scoring function used by Glide (GLIDE SCORE)^64^ can be seen as a modified and expanded version of CHEMSCORE,^96^ herein adopted when software GOLD is used. Furthermore, GOLD and GLIDE differ for the used search algorithm. Indeed, GLIDE employs an algorithm approximating a systematic search of positions, orientations, and conformations of the ligand in the receptor-binding site using a series of hierarchical filters, while GOLD uses a genetic algorithm to explore the full range of ligand conformational flexibility. Finally, differently from GOLD, the docking scores returned by GLIDE include Epik state penalties so that low-populated protonation states are penalized.

### Generation of Protein–Ligand Interaction Fingerprints

In the first step, a common binding site (BS) was defined for all of the investigated compounds using a 9 Å cutoff radius from all atoms of the molecule showing the best docking score. This operation was performed for each model and the interaction fingerprints (IFs) were generated using the SIFt tool available from the Schrodinger Suite 2019-4.^77,97^ Notice that IFs are binary one-dimensional (1D) representations encoding the presence or the absence of specific interactions occurring between a given compound and the BS in the top-scored docking pose. In particular, for each residue belonging to the BS, nine types of possible interactions were considered: (i) any contact, (ii) backbone interactions, (iii) side-chain interactions, (iv) contact with polar residues, (v) contact with hydrophobic residues, (vi) formation of hydrogen bonds with H-bond acceptors of the BS, (vii) formation of hydrogen bonds with H-bond donors of the BS, (viii) contact with aromatic residues, and (ix) contact with charged residues. By doing so, each residue belonging to the BS was represented by a nine-bit long string, where 1 indicates the presence of the corresponding ligand–residue interaction in at least one monomer, and 0 indicates the absence of the same interaction in all of the monomers.

### SVM and LASSO Models

We used, as a first step, the obtained docking scores (DSs) as input for training SVM models.^98^ The performance of the obtained classifiers was evaluated using different quality metrics to identify the protein models more useful to distinguish *hERG* binders from nonbinders. For those classifiers derived using IC_50_ = 80 μM as the inactivity threshold, the area under the ROC curve (AUC)^99^ was computed using the output scores from each SVM model for unseen samples. To provide a DS threshold that corresponds to the separation point between the two classes, the classifier outputs were computed at varying DSs in the range of the observed DS values with a step of 0.01, and the DS value corresponding to the change of the label from active to inactive was recorded. Another aim of our work was to test whether classification models including IFs as additional predictors outperform classifiers based on DS only. Linear classification methods for two-class learning enable to jointly consider associations between DS and the presence or the absence of specific interactions in the IFs and the label of the molecular activity. Linear models with L1-regularization constraint (LASSO) classifiers handle efficiently sparse high-dimensional data structures such as input data consisting of DS and IFs being able to overcome overfitting issues. Models based on these data were trained using LASSO with the SVM learner and the sparsa solver. LASSO is a widely known model introduced by Tibshirani^100^ in which the target value is expected to be a linear combination of the features with an L1-penalty term added to the objective function. To represent both continuous and binary variables in a single vector on which it is possible to apply classification models, our data were preprocessed as follows. DS values were standardized (DSst) according to the following transformation
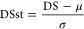
where μ is the mean and σ is the standard deviation on the observed DSs. In the IFs, the values −1 and 1 indicate the absence or the presence of a specific ligand–residue interaction, respectively. The LASSO model tries to set as many coefficients as possible to zero unless a certain residue is really important to drive correctly the predictions. The amount of regularization applied depends on a parameter that takes values in the (0,1) range, and when it takes larger values, the L1-penalty term has a higher weight in the objective function and this leads to an increase in the predictor variable sparsity, namely, fewer interactions will be retained by the model. At varying the regularization strength, a LASSO model was trained and the minimum classification error rate on unseen samples was used to learn the value of the regularization weight. All data analyses were completed in MATLAB using the Statistics and Machine Learning Toolbox (see the Supporting Information for methodological details).

### Evaluation of the Prediction Performance

To evaluate the models’ performance, accuracy (ACC), sensitivity (SE), specificity (SP), and negative predictive values (NPVs) were calculated as follows
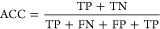

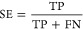

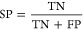

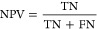
where true positives (TP) and false negatives (FN) are the numbers of known binders predicted to be binders and nonbinders, while true negatives (TN) and false positives (FP) are the numbers of known nonbinders predicted to be nonbinders and binders, respectively.

## Results and Discussion

**Figure 2:**
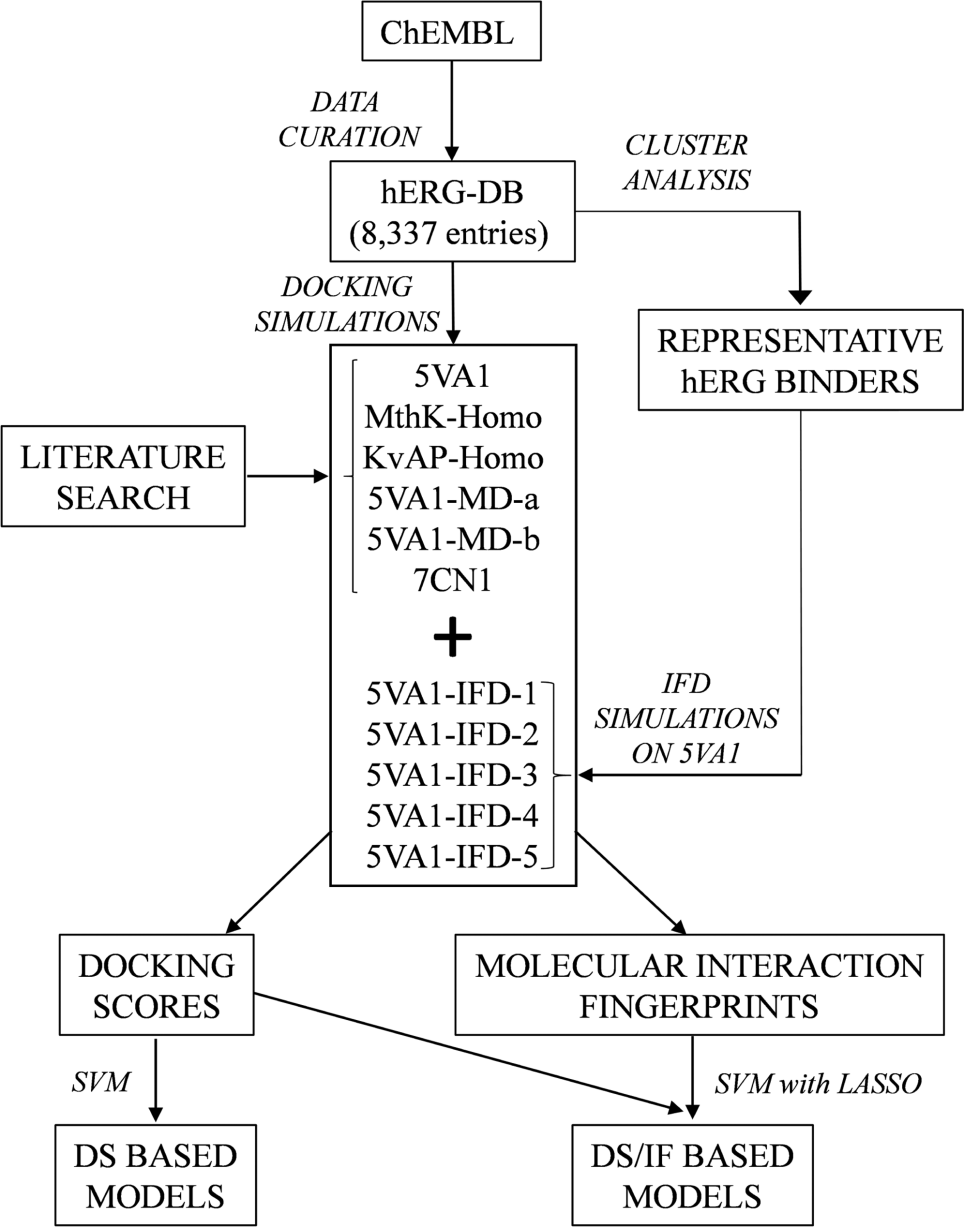
Flowchart showing the main steps of the adopted computational workflow.

For the sake of clarity, a flowchart summarizing the main steps of the adopted computational protocol is reported in Figure 2, while in the following subsections, the obtained results will be presented and discussed. Notice that all of the quality metrics were computed using compounds not included in the training phase, as reported in the “Materials and Methods” section, and that the SE and SP values at varying inactivity thresholds are reported in the Supporting Information (Tables S2 and S3, respectively).

### Evaluation of the Starting Protein Structures

The entire *hERG-DB* was docked into the binding sites of 5*VA*1, *KvAP-Homo*, and *MthK-Homo* to assess the ability of the selected protein structures to generate predictive docking-based classifiers. Notice that, based on mutagenesis studies,^47,89−93^ the protein region including T623, S624, V625, G648, T652, F656, and F557 can be reasonably considered as the *hERG* BS. This is supported by the evidence that this site is relatively larger when compared to the corresponding cavity of other K^+^ channels, consistently with the higher drug promiscuity observed in *hERG*.^55^

In particular, as pointed out in a recent co-authored paper,^14^ an in-depth visual inspection reveals the presence of an atypical BS conformation in 5*VA*1 (Figure S1 in the Supporting Information). Based on that, 5*VA*1 has been widely employed as the structure of choice to perform molecular docking simulations.^44,56−62^ However, such a structural model suffers from two important limitations, which are as follows: (i) it has a resolution (3.7 Å), which is too low to provide an atomic model of the protein and (ii) the model was derived in the absence of a ligand, thus totally neglecting the BS conformational rearrangement occurring upon ligand binding (i.e., induced-fit effects).

In this regard, it should be noted that developing high-quality cryo-EM models accounting for induced fit effects is extremely challenging as the presence of a small molecule in the CC is able to disrupt the *hERG* symmetry, which is required for properly solving the protein structure.^55,75^ In other words, there is no guarantee that this structure is of sufficient quality for reliable docking simulations. Having said that, we performed a preliminary investigation aimed at testing the hypothesis, decisive for the present study, that there are significant differences between hERG binders and nonbinders in terms of the docking score (DS). More specifically, using a Kolmogorov–Smirnov test, we tested the null hypothesis that binders and nonbinder DS values come from populations with the same distribution, against the alternative hypothesis that they are from different distributions. Satisfactorily, very low *p*-values (maximum value equal to 4 × 10^–17^) were obtained for all of the considered protein structures and thresholds (see Table S2 in the Supporting Information). Encouraged by these preliminary data, 54 classifiers were developed using GOLD and GLIDE as software and 5*VA*1, *MthK-Homo*, and *KvAP-Homo* as protein structures and nine different IC_50_ inactivity thresholds (see the Materials and Methods section for methodological details). Notice that when GLIDE was employed as software, the models were derived excluding a small fraction of compounds from the *hERG-DB* [i.e., a percentage from 0.50% (*KvAP-Homo*) to 3.02% (*cryo-EM*) of undocked molecules].

Table 1, reporting the computed accuracies (ACC) for all of the developed classifiers, clearly shows that 5*VA*1 ensures performances (ACC_MAX_ = 0.70 ± 0.01) better than those returned by the homology models herein considered only if GLIDE is used as software. In particular, ACC_MAX_ = 0.62 ± 0.01 and 0.67 ± 0.01 were returned by *MthK-Homo* (KS test *p*-value = 2.2 × 10^–20^) and *KvAP-Homo* (KS test *p*-value = 3 × 10^–6^), respectively. Regarding the classifiers derived using GOLD, both homology models strongly outperform 5*VA*1 (ACC_MAX_ = 0.60 ± 0.01) returning an ACC_MAX_ = 0.73 ± 0.01 (*MthK-Homo* KS test *p*-value = 4 × 10^–34^) and ACC_MAX_ = 0.70 ± 0.01 (*KvAP-Homo* KS test *p*-value = 7 × 10^–29^). In other words, these data suggest that the selection of the protein structure to be used for docking simulations should be performed according to the docking software to be employed. The goodness of the classifiers was also assessed by computing the NPVs, a widely used metric in the context of predictive toxicology^41,42^ as it measures the ability of the model to properly classify nontoxic compounds, namely, to minimize false negatives (i.e., *hERG* binders incorrectly classified as nonbinders). The obtained data are reported in Table 2 showing that, for all of the starting *hERG* structures, the trend discussed based on the computed ACCs is almost confirmed with 5*VA*1, providing the best NPV (NPV_MAX_ = 0.70 ± 0.01) when GLIDE is used as software and the homology models ensuring the best performances when the software employed is GOLD with NPV_MAX_ = 0.74 ± 0.01 (*MthK-Homo*) and NPV_MAX_ = 0.72 ± 0.01 (*KvAP -Homo*).

**Table 1:**
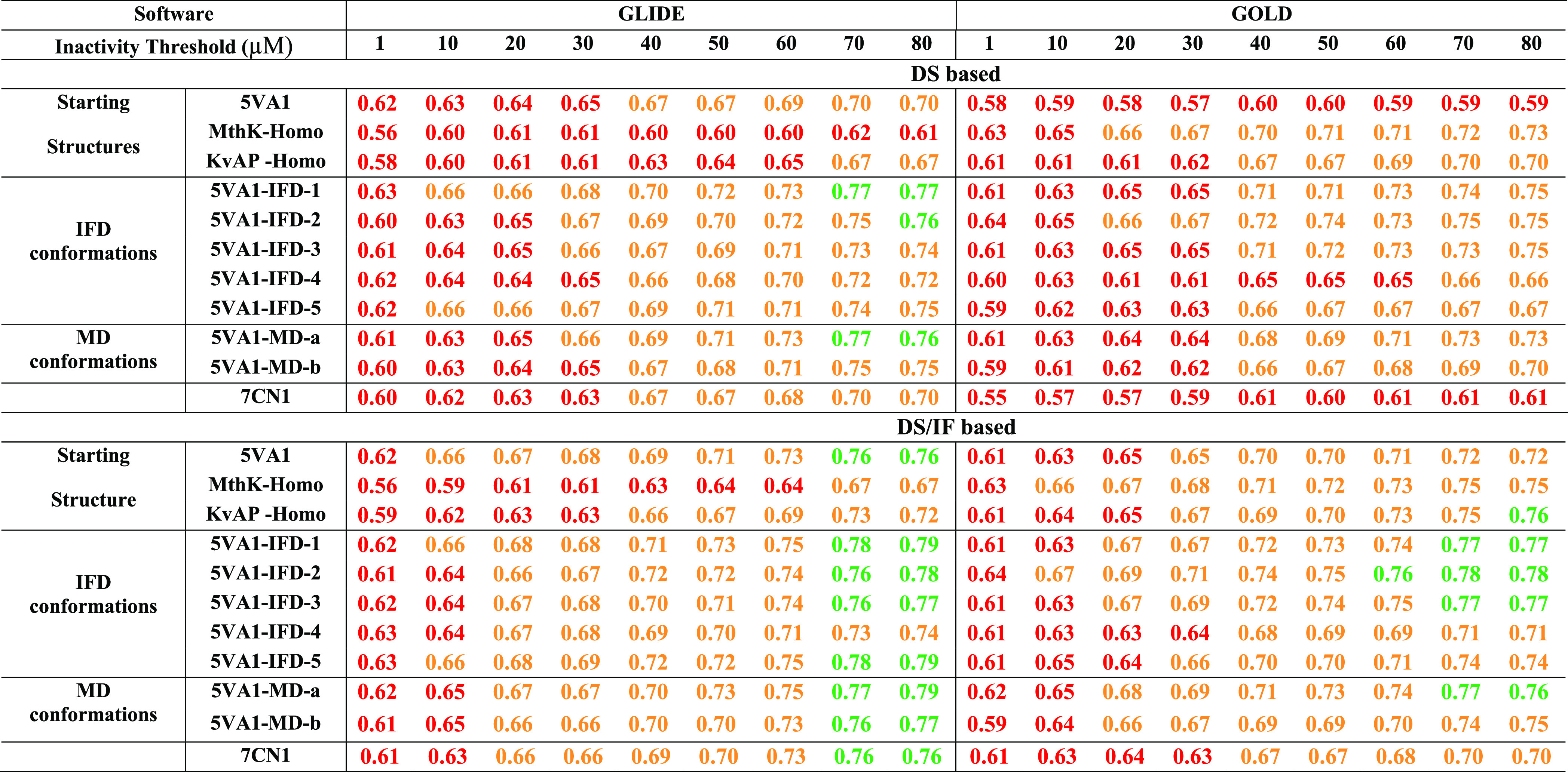
ACCs Returned by the Developed Classifiers on the Basis of Docking Scores (Top) and Docking Scores and IFs (Bottom) Using GLIDE (Left) and GOLD (Right) as Software Programs^a^

a Notice that different inactivity thresholds (μM) were considered, as described in the Materials and Methods section. For the sake of clarity, ACC values >0.50 and ≤0.65, >0.65 and ≤0.75, and >0.75 are reported in red, orange, and green, respectively.

**Table 2:**
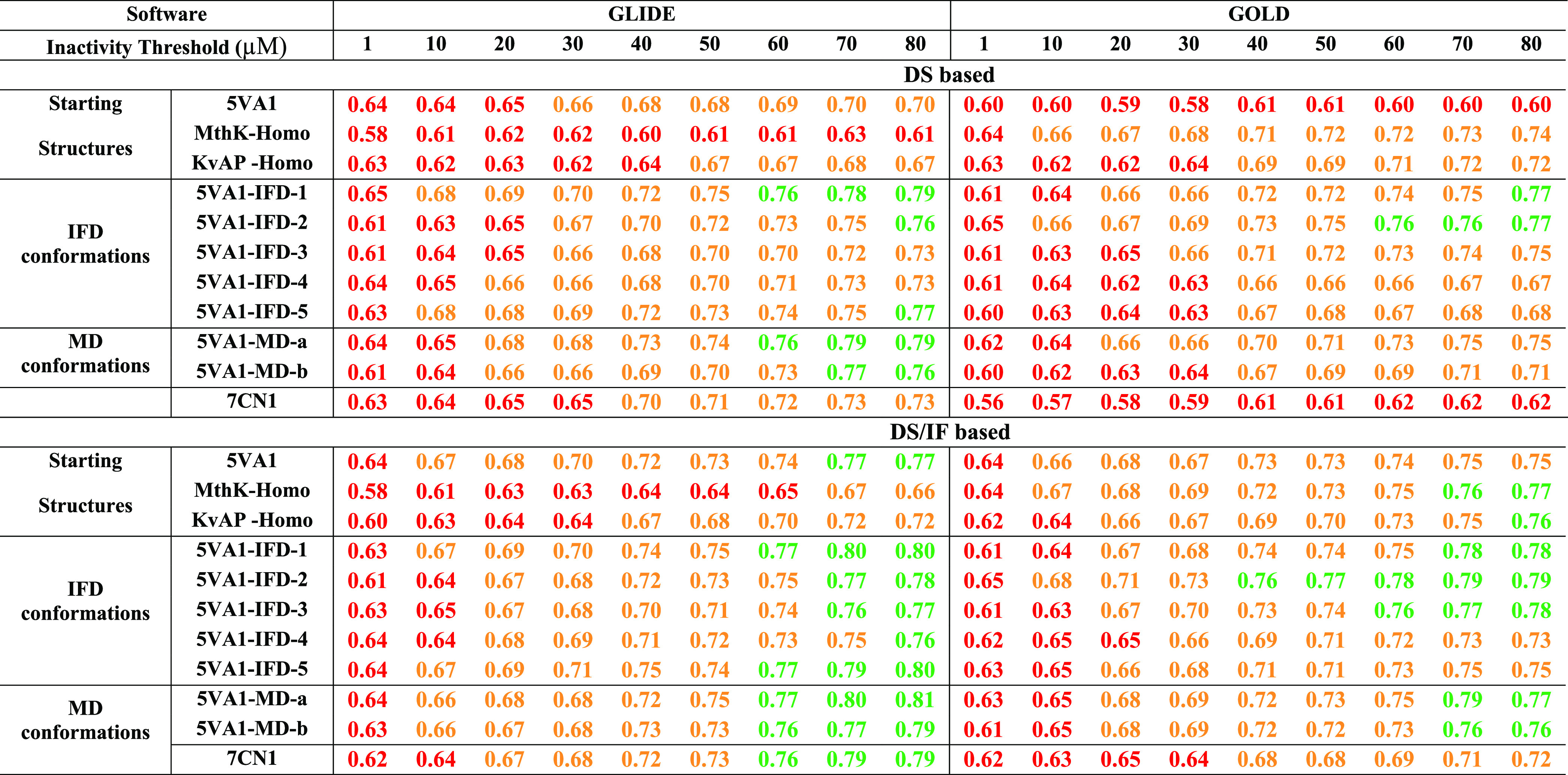
NPVs Computed for All of the Developed Classifiers on the Basis of Docking Scores (Top) and Docking Scores and IFs (Bottom) Using GLIDE (Left) and GOLD (Right) as Software^a^ Programs

a Notice that different inactivity thresholds (μM) were considered, as described in the Materials and Methods section. For the sake of clarity, NPV values >0.50 and ≤ 0.65, > 0.65 and ≤ 0.75, and >0.75 are reported in red, orange, and green, respectively.

Although encouraging in terms of performance, these models were developed based on the DSs only (hereinafter named DS-based models), a strategy commonly employed for developing structure-based classifiers.^41,42^ However, in addition to providing a score estimating the binding affinity, molecular docking simulations predict the conformation as well as the position and orientation of a given ligand (usually referred to as pose) in the target cavity. This piece of information was recently proved to be crucial to overcoming DS deficiencies in virtual screening campaigns.^101−103^ These evidence prompted us to develop classifiers integrating the information provided by both scoring and posing by taking into account the IFs, namely, 1D representations of the ligand–protein interactions occurring in the top-scored docking poses. To this aim, classification models based on sparse high-dimensional data structures consisting of DSs and IFs (hereinafter called DS/IF-based models) were trained using linear models with L1-regularization constraint (LASSO) with the SVM learner and the sparsa solver (see the Materials and Methods section for details). A comparative analysis based on KS tests on the distributions of ACC and NPV values was performed to establish whether DS/IF-based models outperform the DS-based ones. Interestingly, the IFs’ integration allowed obtaining significantly better performances in terms of both ACC (Table 1) and NPV (Table 2), irrespective of the used starting structure. A meaningful example is given by the classifier returned by 5*VA*1 when GLIDE is used as software and 80 μM as inactivity threshold returning ACC (0.76 ± 0.01) and NPV (0.77 ± 0.01) values significantly higher (KS-test *p*-values equal to 1.6 × 10^–^^17^ and 4.6 × 10^–^^18^ for the comparison of ACC and NPV, respectively) than those of the corresponding DS-based model (ACC and NPV = 0.70 ± 0.01). Such an improvement is even more evident when docking simulations are performed on 5*VA*1 with GOLD, as apparent, for instance, looking at the ACC and NPV values returned when 80 μM is used as the inactivity threshold (0.72 vs 0.59, KS-test *p*-value 1.1 × 10^–^^35^ and 0.75 vs 0.60, KS-test *p*-value 4.6 × 10^–31^). These data, taken as a whole, suggest that developing DS/IF-based models can be a winning strategy to develop highly performing classifiers based on docking simulations on the considered *hERG* starting structures.

### Impact of Ligand-Induced Fit Effects on Model Performance

**Figure 3:**
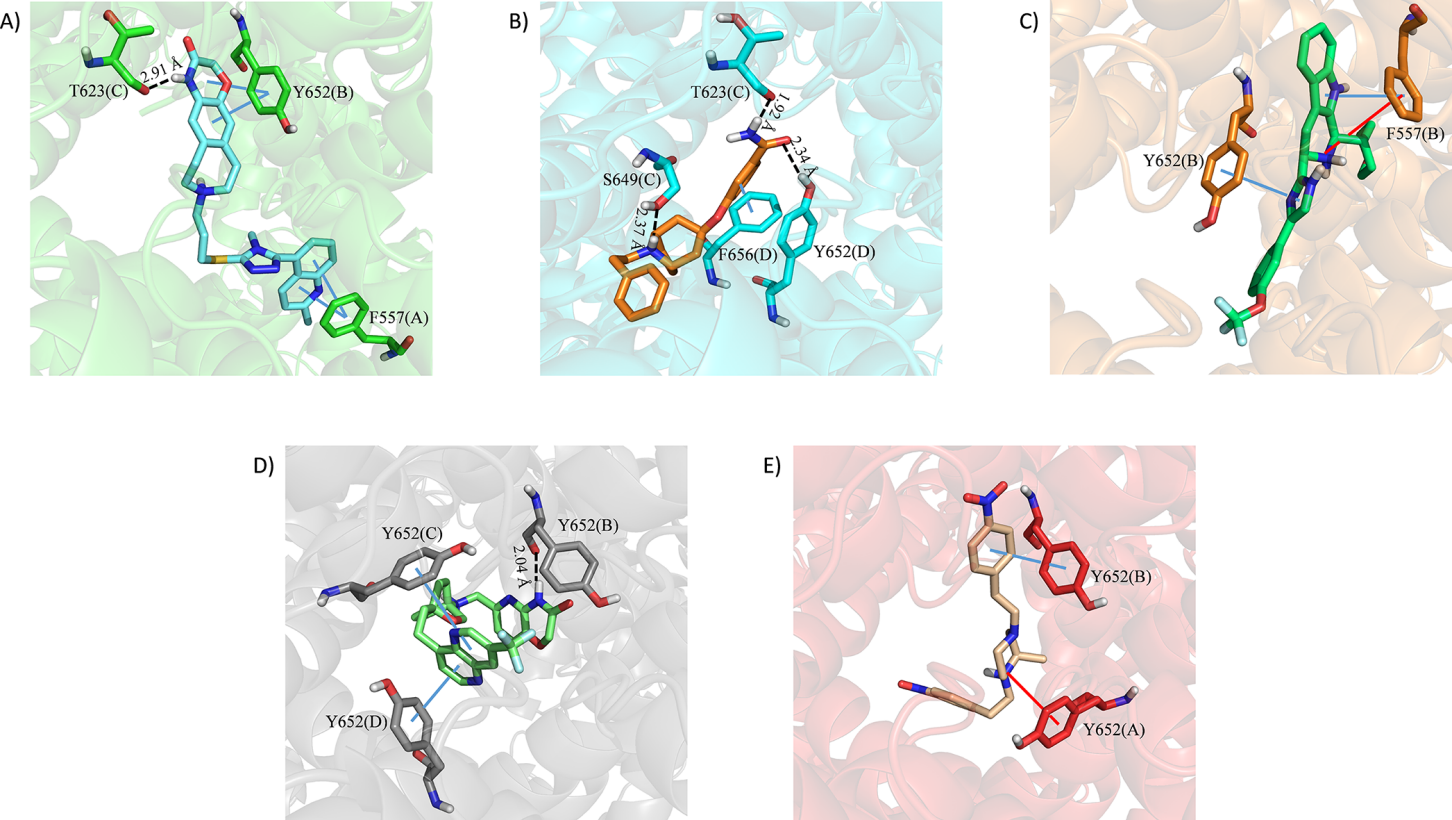
Top-scored docking poses returned by IFD simulations performed on five representative *hERG* binders: (A) CHEMBL271066, (B) CHEMBL1257698, (C) CHEMBL3775456, (D) CHEMBL3422978, and (E) CHEMBL2146867. Ligands and important residues are rendered as sticks, whereas the protein is represented as a cartoon. H-bonds are represented by dotted black lines, whereas the pi-stacking interactions and salt bridge interactions are itemized by a blue and red line, respectively. For the sake of clarity, only polar hydrogen atoms are shown.

As mentioned above, 5*VA*1 was derived in the absence of a ligand, hence no information about the putative BS conformational rearrangement occurring upon ligand binding can be derived from such a structural model. Computational strategies such as IFD and MD simulations are recognized tools for overcoming this limitation, being able to provide the prediction of the BS conformation required for ligand binding. Keeping this in mind, we generated five new *hERG* conformations by performing IFD simulations of five representative and highly affine binders on the 5*VA*1 structure. The resulting top-scored docking poses are depicted in Figure 3.

The obtained protein conformations were named 5*VA*1-*IFD-x*, where *x* refers to the ligand used in the IFD simulation, according to the labeling shown in Figure 1. In addition, we also employed (i) two conformations resulting from MD simulations performed on 5*VA*1 strongly agreeing with mutagenesis data and recently published by Dickson et al.,^44^ as allowing discrimination of blockers vs nonblockers (5*VA*1*-MD-b*) and activators vs nonactivators (5*VA*1-*MD-a*) and (ii) an *hERG* model published at the time of writing the present paper and obtained through electron microscopy in the presence of the known blocker astemizole (PDB code 7CN1).^75^ All of these BS conformations, depicted in Figure S2, were therefore employed to derive 288 (144 DS-based and 144 DS/IF-based) classifiers by taking into account again nine different IC_50_ inactivity thresholds and GLIDE and GOLD as software. The obtained ACC and NPV values are reported in Tables 1 and 2, respectively. Interestingly, the use of both IFD- and MD-based protein conformations allowed obtaining much more performing classifiers than the starting 5*VA*1 model. The improvement observed in the DS-based classifiers is worth noting: all of the new conformations provide higher ACC and NPV values for inactivity thresholds ≥50 μM in the case of GLIDE used as software and for all of the inactivity thresholds when GOLD is employed. Notably, 7*CN*1 was responsible for performances in line with those returned by 5*VA*1, in agreement with the picture emerged from a three-dimensional comparison of the two structures (data not shown), indicating the presence of very similar binding pockets.

In other words, albeit obtained using electron microscopy experiments performed in the presence of a blocker, this protein conformation is outperformed by those derived by computational procedures as IFD and MD. More specifically, the best performances are ensured by 5*VA*1-*IFD-*1 (ACC_MAX_ = 0.77 ± 0.01 and NPV_MAX_ = 0.79 ± 0.01) and 5*VA*1*-MD-a* (ACC_MAX_ = 0.77 ± 0.01 and NPV_MAX_ = 0.79 ± 0.01) if the software employed is GLIDE, as well as 5*VA*1*-IFD-*1 and 5*VA*1*-IFD-*2 (ACC_MAX_ = 0.75 ± 0.01 and NPV_MAX_ = 0.77 ± 0.01 for both) when GOLD is used. It is worth noting that the homology models used as starting structures are also outperformed by most of the IFD and MD conformations. As far as the DS/IF-based classifiers are concerned, such a trend is confirmed with the best performances returned by 5*VA*1*-IFD-*1 (ACC_MAX_ = 0.79 ± 0.01 and NPV_MAX_ = 0.80 ± 0.01), 5*VA*1*-IFD-*5 (ACC_MAX_ = 0.79 ± 0.01 and NPV_MAX_ = 0.80 ± 0.01), and 5*VA*1*-MD-a* (ACC_MAX_ = 0.79 ± 0.01 and NPV_MAX_ = 0.81 ± 0.01) after using GLIDE and 5*VA*1*-IFD-*2 (ACC_MAX_=0.78 ± 0.01 and NPV_MAX_ = 0.79 ± 0.01) when GOLD is employed. Notice that significantly worst performances were returned by both 5*VA*1 and 7*CN*1 structures. It is worth noting that, as already observed for the starting structures, also for the 5*VA*1*-IFD-x* protein conformations, DS/IF-based models (ACC_MAX_ = 0.79 ± 0.01 and 0.78 ± 0.01 using GLIDE and GOLD, respectively) outperform DS-based ones (ACC_MAX_ = 0.77 ± 0.01, KS-test *p*-value = 0.07, and 0.75 ± 0.01, KS-test *p*-value = 0.004) using GLIDE and GOLD, respectively, in terms of ACC.

### Selection of the Best-Performing *hERG* Conformation

**Figure 4:**
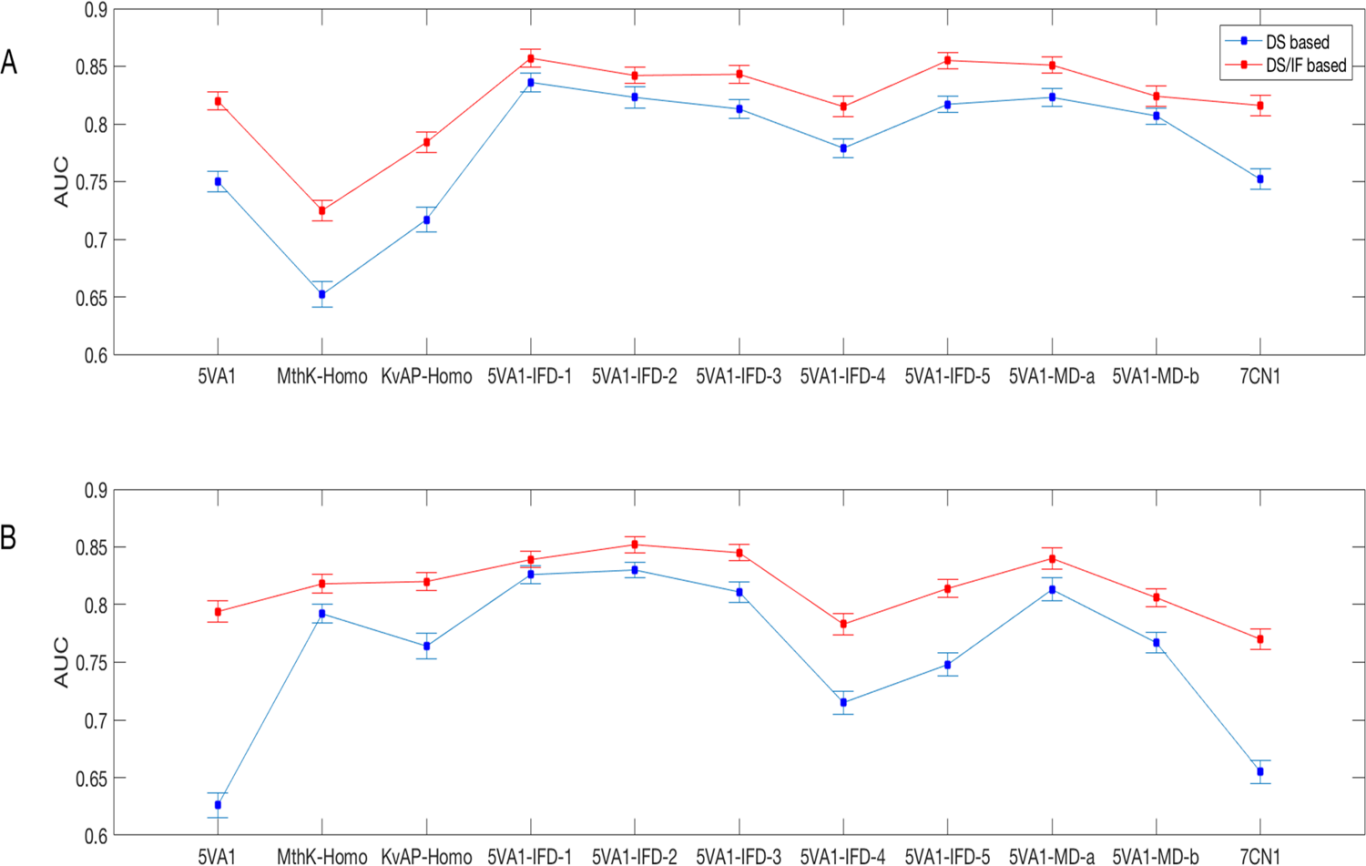
Two-dimensional (2D) plot reporting the AUC values computed for the classifiers developed using IC_50_ = 80 μM as the inactivity threshold and (A) GLIDE and (B) GOLD as software programs.

The picture emerged from the discussed data suggests that the best-performing classifiers are those developed accounting for ligand-induced fit effects. However, based on the considered quality metrics, it is still hard to select the best BS conformation to be used for docking simulations. To make a final selection, we also computed the area under the ROC curve (AUC) for all of the classifiers developed using IC_50_ = 80 μM as the inactivity threshold, being those ensuring the greatest performances irrespective of the considered software program and methodology (DS and DS/IF-based). Figure 4 reports a plot of the computed AUC values for different protein conformations.

Remarkably, DS/IF-based models significantly outperform DS-based ones (KS *p*-values < 0.05), irrespective of the employed protein conformation and the software program with the best performances obtained by 5*VA*1*-IFD-*1 (AUC = 0.86 ± 0.01), 5*VA*1*-IFD-*5 (AUC = 0.86 ± 0.01), and 5*VA*1*-MD-a* (AUC = 0.85 ± 0.01) when GLIDE is used and 5*VA*1*-IFD-*2 (AUC = 0.85 ± 0.01), 5*VA*2*-IFD-*3 (AUC = 0.85 ± 0.01), and 5*VA*1*-MD-a* (AUC = 0.84 ± 0.01) if GOLD is employed. Furthermore, when conformations accounting for ligand-induced fit effects are taken into account, satisfactory AUC values are computed even without the IF integration with the best performances ensured by 5*VA*1*-IFD-*1 (AUC = 0.84 ± 0.01) when using GLIDE and both 5*VA*1*-IFD-*1 (AUC = 0.83 ± 0.01) and 5*VA*1*-IFD-*2 (AUC = 0.83 ± 0.01) in the case of GOLD employed as a software program. It is worh noting that although from a methodological point of view, it should remarked that the IF integration allows obtaining better performances, models based on DS only should be preferred from a practical point of view, especially when developed using highly performing *hERG* protein models such as 5*VA*1*-IFD-*1. Indeed, DS-based classifiers are characterized by higher interpretability than DS/IF ones and can be employed by interested users by simply comparing the docking scores returned by the chemicals of interest with the DS thresholds reported in Table 3.

**Table 3:** DS Thresholds for All of the DS-Based Models Developed Using 80 μM as the IC_50_ Inactivity Threshold. Notice that the DSs are Expressed by kcal/mol and kJ/mol, as Returned by the Software Programs GLIDE and GOLD, Respectively

	GLIDE		GOLD	
hERG conformation	DS threshold (kcal/mol)	standard deviation	DS threshold (kJ/mol)	standard deviation
5VA1	–6.012	±0.003	–25.989	±0.023
MthK-Homo	–5.140	±0.003	–30.792	±0.016
KvAP-Homo	–5.659	±0.003	–28.162	±0.012
5VA1-IFD-1	–8.967	±0.004	–37.444	±0.011
5VA1-IFD-2	–7.790	±0.004	–34.812	±0.016
5VA1-IFD-3	–8.131	±0.004	–34.713	±0.013
5VA1-IFD-4	–7.063	±0.004	–28.768	±0.015
5VA1-IFD-5	–7.068	±0.003	–30.002	±0.013
5VA1-MD-a	–8.472	±0.003	–37.384	±0.019
5VA1-MD-b	–8.349	±0.003	–34.376	±0.013
7CN1	–6.010	±0.004	–28.807	±0.019

It is worth noting that based on the discussed data, 5*VA*1*-IFD-*1 can be reasonably considered as the *hERG* conformation of choice for reliable docking simulations, and for this reason, was made available, along with the other 5VA1-IFD conformations, in the Supporting Information as a. pdb file. Remarkably, 5VA1-IFD-1 is also the conformation returning the highest BS volume (789.56 Å^3^), as reported in Table S5. Based on this, it is reasonable to speculate that the larger the *hERG* BS, the higher the ability, during the performed docking simulations, to properly accommodate compounds with very different shapes and sizes as those belonging to the *hERG-DB*.

### IF-Based Analysis

Encouraged by the ability of the computed IFs to improve classifiers’ performance, we conducted an in-depth IFs analysis aimed to get insights into the structural basis for high-affinity *hERG*–drug binding. To identify key protein–ligand interactions, the distributions of the IC_50_ values of compounds interacting/noninteracting with a specific residue (1/0 in the interaction fingerprint respectively) were investigated using KS tests that allowed us to identify the interactions responsible for a significantly lower value of IC_50_. In particular, we performed the test 100 times for each residue on compounds randomly drawn from the entire set of molecules to distinguish general findings not specific for subsets of molecules. We focused our attention on the IFs returned by the best-performing conformation, namely, 5*VA*1*-IFD-*1. Table 4 shows the residues sorted by the number of occurrences of significant KS test *p*-values (*p* < 0.05) in the 100 trials (the occurrence is shown in square brackets). The interested reader is referred to Table S6 for data returned by all of the *hERG* protein models. In particular, as evident in Table 4, some interactions established with the side chains of F557 (hydrophobic and aromatic), M651 (hydrophobic), I655 (hydrophobic), and F656 (hydrophobic and aromatic) were predicted to be crucial, being detected with the highest number of occurrences of significant *p*-values irrespective of the employed software program. It is worth noting that the obtained data are in agreement with experimental findings, mostly based on alanine-scanning mutagenesis. F656, for instance, was proved to be crucial for the blocking ability of cisapride by Chen et al.,^104^ while several mutagenesis studies^67,89^ emphasized the importance of F557 in the *hERG* recognition of different drugs. Finally, Kudaibergenova et al. in a paper published in 2020 and reporting experimental data returned by a mutant (i.e., M651T),^105^ put forward, for the first time, M651 as another key residue for *hERG*–drug binding.

**Table 4:** Interactions Responsible for a Lower IC_50_ Based on the KS Test Performed on the IFs Returned by 5VA1-IFD-1

GLIDE	GOLD
557_aromatic[100]	554_contact[100]
557_contact[100]	557_aromatic[100]
557_hydrophobic[100]	557_contact[100]
557_sidechain[100]	557_hydrophobic[100]
649_backbone[100]	557_sidechain[100]
655_contact[100]	648_contact[100]
655_hydrophobic[100]	648_sidechain[100]
655_sidechain[100]	649_polar[100]
656_backbone[100]	649_sidechain[100]
649_contact[98]	651_backbone[100]
651_hydrophobic[98]	651_contact[100]
651_sidechain[98]	655_contact[100]
652_backbone[93]	655_hydrophobic[100]
656_contact[91]	655_sidechain[100]
651_backbone[89]	656_aromatic[100]
656_aromatic[89]	656_backbone[100]
656_hydrophobic[89]	656_contact[100]
656_sidechain[89]	656_hydrophobic[100]
651_contact[89]	656_sidechain[100]
652_aromatic[32]	554_hydrophobic[99]
652_hydrophobic[32]	554_sidechain[99]
652_sidechain[32]	649_backbone[99]
649_polar[28]	649_contact[99]
649_sidechain[28]	655_backbone[99]
653_hydrophobic[25]	652_backbone[98]
653_sidechain[25]	651_hydrophobic[78]
655_backbone[14]	651_sidechain[78]
653_contact[9]	659_contact[66]
553_backbone[7]	659_hydrophobic[66]
553_contact[7]	659_sidechain[66]
623_backbone[5]	553_backbone[28]
	553_contact[28]
	650_contact[1]

## Conclusions

In this work, we trained the first structure-based models of *hERG*-related cardiotoxicity based on bioactivity data reported in ChEMBL (version 25) and both docking scores and protein–ligand interaction fingerprints returned by the software programs GLIDE and GOLD for different protein structures used as *hERG* structural models, including those recently obtained through cryoelectron microscopy (PDB codes: 5VA1^55^ and 7CN1^75^). A total of 396 models were built based on the support vector machine and the LASSO regularized support vector machine and evaluated using different quality metrics (i.e., ACC, NPV, and AUC). Remarkably, some models returned performances comparable to ligand-based classifiers,^29,33,35−37^ whose usage is often limited by their restricted applicability domain and low interpretability. Finally, based on a comparative analysis of all of the derived classifiers, we concluded that the integration of docking scores and molecular interaction fingerprints is a winning strategy to maximize model performance, as the proposed method outperforms that based on docking scores only. Importantly, much more reliable docking-based predictions are obtained using a new protein conformation returned by IFD simulations (made available in the Supporting Information as a. pdb file) instead of the cryo-EM model, as it is (i.e., PDB code: 5VA1^55^), which is the usual practice.^44,56−62^ From a methodological point of view, the study represents the first attempt to incorporate the information provided by docking poses in structure-based classifiers using a LASSO SVM regularized strategy thus providing a new computational workflow to be used in the context of predictive toxicology.

## Supporting Information Available

Top views of the BS of all of the employed protein models and the number of active and inactive compounds as a consequence of the selected activity and inactivity thresholds (Figures S1 and S2 and Tables S1) (PDF)
Kolmogorov–Smirnov test *p*-values summarizing the difference in docking score distributions between hERG binders and nonbinders, the SE and SP of all of the developed models, the BS volumes (Å^3^) of all of the used hERG models, and the interactions responsible for a lower IC_50_ based on the KS test performed on the IFs returned by all of the considered protein models (Tables S2–S6) (PDF)
List of the compounds belonging to the *hERG-DB*, including SMILES strings and corresponding IC_50_ values (XLSX)
Results of the performed docking simulations including docking scores and molecular interaction fingerprints (ZIP)
Results of the performed docking simulations including molecular interaction fingerprints (ZIP)
5VA1-IFD-1, 5VA1-IFD-2, 5VA1-IFD-3, 5VA1-IFD-4, and 5VA1-IFD-5 conformations as. pdb files (ZIP)
