# Supplementary material for: Structure-Based Prediction of hERG-Related Cardiotoxicity: A Benchmark Study
Source: J Chem Inf Model. 2021 Sep 10;61(9):4758–70. doi: 10.1021/acs.jcim.1c00744 (PMC9282647; doi:10.1021/acs.jcim.1c00744)
Supplement: Supplementary file 1 — ci1c00744_si_001.pdf [file ci1c00744_si_001.pdf]

Structure-based prediction of hERG-related cardiotoxicity: a benchmark study

Teresa Maria Creanza[a, Ω], *Pietro Delre*[b,c, Ω], *Nicola Ancona*[a], *Giovanni Lentini*[d], *Michele Saviano*[c] *and Giuseppe Felice Mangiatordi*[c]*

a CNR - Institute of Intelligent Industrial Technologies and Systems for Advanced Manufacturing, Via Amendola 122/o, 70126 Bari, Italy

b Chemistry Department, University of Bari "Aldo Moro", via E. Orabona, 4, I-70125 Bari, Italy.

c CNR – Institute of Crystallography, Via Amendola 122/o, 70126 Bari, Italy

d Department of Pharmacy - Pharmaceutical Sciences, University of Bari "Aldo Moro", via E. Orabona, 4, I-70125 Bari, Italy

*Correspondence: giuseppe.mangiatordi@ic.cnr.it (G.F.M.); +39-080-5929158 (G.F.M.);

[Ω]: These authors contributed equally to this study.

Supplementary material - Table of content

Details on data processing

Figure S1. Top view of the A) *5VA1*; B) *KvAP -Homo* and C) *MthK-Homo* BS. Key residues are depicted as sticks and named for one monomer only. For the sake of clarity, only polar hydrogen atoms are shown.

Figure S2. Top view of the A) *5VA1-IFD-1*; B) *5VA1-IFD-2*; C) *5VA1-IFD-3*; D) *5VA1-IFD-4*; E) *5VA1-IFD-5*; F) *5VA1-MD-a*; G) *5VA1-MD-b* and H) *7CN1* BS. Key residues are depicted as sticks and named for one monomer only. For the sake of clarity, only polar hydrogen atoms are shown.

Table S1. Number of active and inactive compounds as a consequence of the selected activity (IC50 = 1μm) and inactivity (IC_{50} = 1μM, 10 μM, 20 μM, 30 μM, 40 μM, 50 μM, 60 μM, 70 μM and 80 μM) thresholds.

Table S2. Kolmogorov-Smirnov test P-values summarizing the difference in docking score distributions between *hERG* binders and non-binders.

Table S3. Sensitivity of all the developed classifiers on the basis of docking scores (top) and docking scores and IFs (bottom) using GLIDE (left) and GOLD (right) as software. Notice that different thresholds (μM) were considered as described in the "materials and methods" section.

Table S4. Specificity computed for all the developed classifiers on the basis of docking scores (top) and docking scores and Ifs (bottom) using GLIDE (left) and GOLD (right) as software. Notice that different thresholds (μM) were considered as described in the "materials and methods" section.

Table S5. Binding site volumes ($Å^3$) computed by SiteMap (Schrodingher Suite 2019-4) for all the 3D hERG models used in this study.

Table S6. Interactions responsible for a lower IC_{50} based on a KS test performed on the IFs returned by all the considered protein models. The table shows the interactions sorted by the number of occurrences of significant KS test p-values ($p < 0.05$) in the 100 trials (the occurrence is shown in square brackets).

Details on data processing

All data analysis was completed in MATLAB using the Statistics and Machine Learning Toolbox. In particular, the function kstest2 was used to perform the Kolmogorov-Smirnov test in order to compare two sample distributions. The function fitcsvm was used to train support vector machine model for two-class classification based on docking score values of hERG binders and nonbinders (low-dimensional predictor data set). In order to process docking scores and protein-ligand interaction fingerprints, the function fitclinear was used to train regularized support vector machines for binary learning with high-dimensional and sparse predictor data. Fitclinear minimizes the objective function using stochastic gradient descent to reduce computing time.

Figure S1

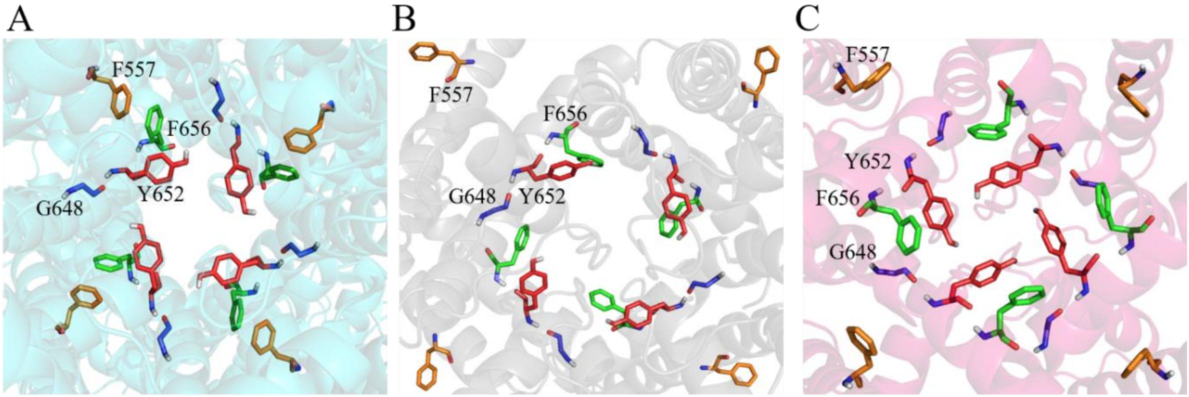

A
F557
F656
G648
Y652
B
F557
F656
G648
Y652
C
F557
Y652
F656
G648

Figure S2

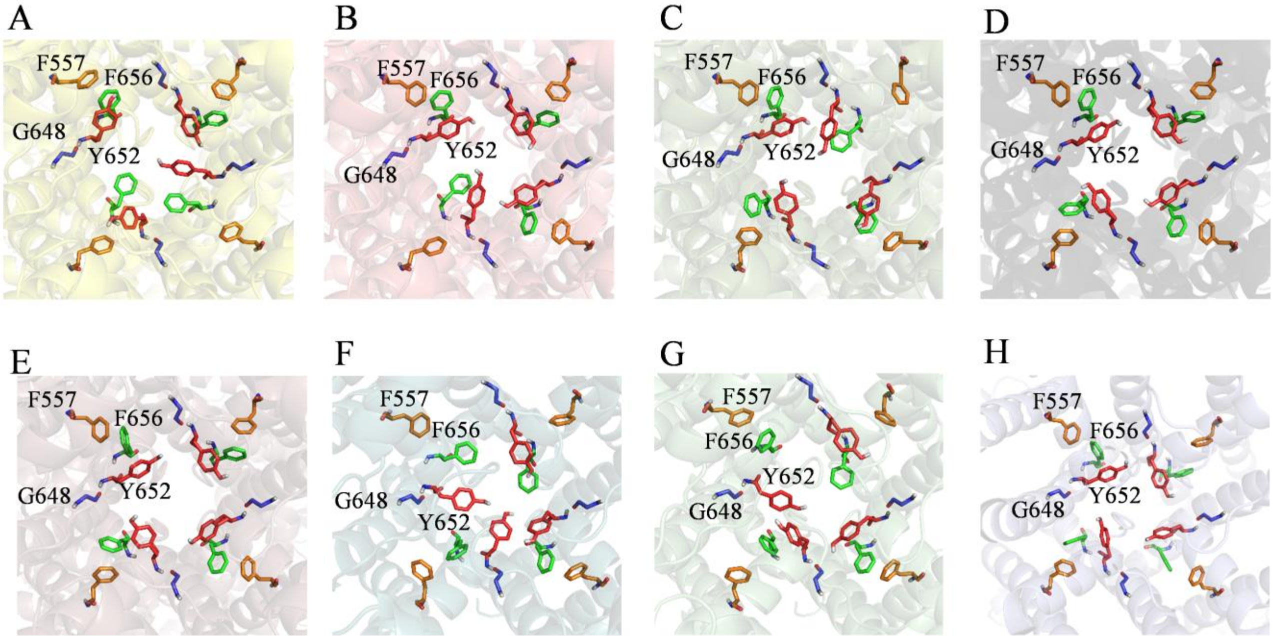
A
F557
F656
G648
Y652
B
F557
F656
G648
Y652
C
F557
F656
G648
Y652
D
F557
F656
G648
Y652
E
F557
F656
G648
Y652
F
F557
F656
G648
Y652
G
F557
F656
Y652
G648
H
F557
F656
G648
Y652

Table S1

	Active compounds	Inactive compounds								
**Thresholds (IC_{50})**	1µM	1µM	10µM	20µM	30µM	40µM	50µM	60µM	70µM	80µM
**Number of molecules**	1308	6782	4127	2822	2326	1384	1242	1078	925	868

Table S2

Threshold (μM)		1	10	20	30	40	50	60	70	80
**GLIDE**										
**Starting Structures**	**5VA1**	6.00E-49	7.46E-71	4.31E-67	6.31E-71	2.48E-62	9.62E-65	1.26E-74	3.11E-75	2.84E-76
	**MthK-Homo**	1.54E-20	7.08E-34	1.71E-31	6.11E-34	5.90E-27	3.57E-27	1.31E-29	2.91E-30	5.53E-29
	**KvAP -Homo**	6.17E-27	1.00E-38	2.19E-38	6.92E-41	4.77E-41	4.84E-43	1.15E-47	9.54E-53	1.55E-51
**IFD conformations**	**5VA1-IFD-1**	7.45E-59	1.81E-82	2.00E-89	3.35E-94	3.75E-100	1.69E-105	3.45E-121	5.05E-139	5.64E-138
	**5VA1-IFD-2**	2.99E-47	2.61E-73	4.70E-83	5.75E-90	2.26E-94	7.54E-98	4.17E-112	1.14E-129	7.31E-129
	**5VA1-IFD-3**	1.94E-48	6.09E-71	1.98E-78	6.96E-84	1.60E-79	5.77E-84	9.92E-98	7.61E-118	7.99E-119
	**5VA1-IFD-4**	5.57E-45	6.17E-68	7.86E-73	7.24E-77	9.56E-69	4.19E-73	8.84E-84	3.30E-90	2.07E-89
	**5VA1-IFD-5**	6.39E-60	1.22E-85	5.69E-89	1.37E-96	6.54E-88	1.04E-91	1.60E-101	1.76E-113	1.28E-118
**MD conformations**	**5VA1-MD-a**	2.31E-43	4.62E-63	3.86E-73	8.75E-81	5.38E-94	8.70E-103	3.40E-117	5.03E-136	2.36E-135
	**5VA1-MD-b**	4.94E-40	1.02E-59	3.59E-65	3.94E-72	1.27E-78	3.71E-84	1.44E-98	8.90E-116	3.95E-116
	**7CN1**	3.57E-35	1.71E-56	9.97E-57	5.82E-62	1.07E-64	2.81E-66	2.81E-71	4.23E-80	2.51E-77
**GOLD**										
**Starting Structure**	**5VA1**	1.67E-24	5.78E-33	3.55E-26	2.29E-24	3.11E-27	1.33E-27	4.57E-25	1.70E-20	1.15E-18
	**MthK-Homo**	9.45E-65	4.65E-90	3.57E-90	3.20E-88	1.61E-99	5.41E-100	1.30E-102	1.25E-99	3.43E-96
	**KvAP -Homo**	8.30E-41	1.14E-44	2.55E-46	6.65E-43	1.18E-66	6.01E-67	1.15E-70	7.75E-83	3.15E-81
**IFD conformations**	**5VA1-IFD-1**	5.81E-44	6.55E-67	4.98E-77	1.57E-78	2.77E-105	5.30E-109	5.93E-111	1.21E-122	8.27E-122
	**5VA1-IFD-2**	1.00E-67	1.69E-90	4.52E-97	6.40E-100	3.49E-128	1.97E-134	1.13E-127	8.71E-131	2.68E-131
	**5VA1-IFD-3**	1.47E-56	1.09E-78	1.30E-82	7.47E-81	2.04E-104	3.17E-111	1.55E-109	5.43E-117	2.10E-115
	**5VA1-IFD-4**	2.22E-41	2.22E-56	5.84E-48	1.83E-45	1.62E-55	8.98E-56	5.71E-57	6.71E-56	4.63E-54
	**5VA1-IFD-5**	5.43E-45	2.32E-62	2.41E-59	2.44E-57	5.44E-73	2.84E-73	1.97E-65	1.17E-64	8.26E-62
**MD conformations**	**5VA1-MD-a**	2.39E-47	3.08E-63	9.08E-72	4.99E-73	2.56E-96	5.48E-99	2.42E-104	1.31E-113	5.93E-113
	**5VA1-MD-b**	5.03E-32	4.53E-45	2.08E-45	3.00E-43	1.94E-65	1.24E-67	1.54E-68	1.94E-75	4.63E-76
	**7CN1**	6.53E-18	9.67E-24	9.67E-24	2.01E-20	1.68E-30	3.00E-30	3.68E-29	9.00E-30	6.59E-28

Table S3

Software		GLIDE									GOLD								
**Inactivity threshold (μM)**		**1**	**10**	**20**	**30**	**40**	**50**	**60**	**70**	**80**	**1**	**10**	**20**	**30**	**40**	**50**	**60**	**70**	**80**
**DSs based**																			
**Starting Structures**	**5VA1**	0.70	0.69	0.67	0.68	0.71	0.72	0.70	0.70	0.71	0.68	0.70	0.74	0.77	0.72	0.74	0.77	0.76	0.74
	**MthK-Homo**	0.66	0.63	0.63	0.61	0.56	0.58	0.57	0.59	0.57	0.68	0.70	0.71	0.72	0.73	0.72	0.74	0.76	0.76
	**KvAP -Homo**	0.58	0.60	0.60	0.60	0.60	0.59	0.61	0.62	0.59	0.71	0.72	0.74	0.75	0.75	0.78	0.78	0.78	0.79
**IFD conformations**	**5VA1-IFD-1**	0.71	0.73	0.74	0.75	0.78	0.80	0.79	0.80	0.79	0.70	0.72	0.74	0.74	0.79	0.79	0.81	0.81	0.82
	**5VA1-IFD-2**	0.69	0.69	0.71	0.72	0.76	0.76	0.75	0.76	0.77	0.75	0.76	0.79	0.81	0.81	0.81	0.82	0.83	0.82
	**5VA1-IFD-3**	0.65	0.64	0.66	0.65	0.71	0.73	0.77	0.75	0.76	0.74	0.73	0.75	0.78	0.78	0.78	0.80	0.81	0.81
	**5VA1-IFD-4**	0.71	0.71	0.74	0.74	0.76	0.77	0.74	0.75	0.76	0.68	0.68	0.68	0.71	0.70	0.72	0.74	0.77	0.76
	**5VA1-IFD-5**	0.70	0.71	0.74	0.74	0.77	0.76	0.77	0.78	0.80	0.63	0.63	0.65	0.63	0.67	0.68	0.69	0.71	0.71
**MD conformations**	**5VA1-MD-a**	0.70	0.71	0.75	0.76	0.83	0.83	0.83	0.83	0.84	0.71	0.75	0.78	0.80	0.84	0.85	0.85	0.87	0.86
	**5VA1-MD-b**	0.66	0.68	0.69	0.70	0.80	0.79	0.81	0.79	0.80	0.67	0.69	0.72	0.73	0.73	0.71	0.72	0.74	0.73
	**7CN1**	0.77	0.75	0.77	0.78	0.80	0.81	0.80	0.81	0.80	0.71	0.68	0.73	0.75	0.71	0.69	0.73	0.74	0.73
**DSs/IFs based**																			
**Starting Structure**	**5VA1**	0.68	0.68	0.70	0.72	0.75	0.76	0.76	0.78	0.78	0.73	0.72	0.73	0.71	0.76	0.77	0.77	0.77	0.77
	**MthK-Homo**	0.70	0.67	0.68	0.67	0.67	0.66	0.66	0.66	0.65	0.67	0.68	0.70	0.72	0.73	0.75	0.77	0.78	0.79
	**KvAP -Homo**	0.64	0.65	0.67	0.67	0.69	0.68	0.71	0.72	0.71	0.64	0.65	0.67	0.68	0.69	0.71	0.73	0.75	0.77
**IFD conformations**	**5VA1-IFD-1**	0.67	0.71	0.72	0.73	0.77	0.78	0.79	0.81	0.81	0.64	0.65	0.69	0.69	0.76	0.76	0.76	0.78	0.79
	**5VA1-IFD-2**	0.61	0.65	0.68	0.69	0.73	0.74	0.76	0.77	0.79	0.68	0.70	0.73	0.74	0.78	0.78	0.80	0.80	0.80
	**5VA1-IFD-3**	0.65	0.66	0.68	0.68	0.71	0.71	0.75	0.76	0.77	0.63	0.63	0.67	0.71	0.74	0.74	0.77	0.78	0.78
	**5VA1-IFD-4**	0.67	0.66	0.70	0.70	0.73	0.75	0.74	0.76	0.77	0.67	0.68	0.68	0.70	0.71	0.73	0.75	0.75	0.75
	**5VA1-IFD-5**	0.68	0.68	0.72	0.73	0.77	0.77	0.79	0.80	0.81	0.67	0.67	0.68	0.70	0.72	0.72	0.74	0.76	0.77
**MD conformations**	**5VA1-MD-a**	0.70	0.70	0.70	0.70	0.74	0.77	0.79	0.81	0.82	0.66	0.65	0.70	0.70	0.72	0.74	0.75	0.80	0.78
	**5VA1-MD-b**	0.69	0.69	0.70	0.71	0.77	0.76	0.79	0.79	0.80	0.65	0.67	0.71	0.73	0.76	0.75	0.76	0.78	0.77
	**7CN1**	0.68	0.67	0.71	0.72	0.75	0.77	0.79	0.81	0.81	0.66	0.65	0.67	0.66	0.69	0.69	0.71	0.73	0.74

Table S4

Software		GLIDE									GOLD								
**Inactivity Threshold (μM)**		**1**	**10**	**20**	**30**	**40**	**50**	**60**	**70**	**80**	**1**	**10**	**20**	**30**	**40**	**50**	**60**	**70**	**80**
**DSs based**																			
**Starting Structures**	**5VA1**	0.52	0.60	0.61	0.62	0.63	0.62	0.67	0.70	0.70	0.45	0.45	0.43	0.41	0.46	0.48	0.42	0.42	0.43
	**MthK-Homo**	0.48	0.55	0.56	0.59	0.64	0.64	0.65	0.65	0.67	0.58	0.62	0.61	0.63	0.67	0.67	0.70	0.68	0.70
	**KvAP -Homo**	0.56	0.61	0.61	0.62	0.64	0.65	0.68	0.72	0.74	0.50	0.49	0.47	0.47	0.56	0.57	0.58	0.63	0.63
**IFD conformations**	**5VA1-IFD-1**	0.54	0.57	0.59	0.59	0.61	0.63	0.67	0.74	0.74	0.50	0.55	0.57	0.59	0.63	0.63	0.64	0.69	0.68
	**5VA1-IFD-2**	0.54	0.58	0.61	0.62	0.64	0.65	0.69	0.75	0.75	0.51	0.55	0.55	0.55	0.65	0.68	0.66	0.69	0.70
	**5VA1-IFD-3**	0.57	0.63	0.65	0.67	0.63	0.64	0.67	0.72	0.73	0.49	0.53	0.57	0.55	0.63	0.67	0.64	0.67	0.68
	**5VA1-IFD-4**	0.49	0.54	0.58	0.57	0.57	0.58	0.63	0.67	0.68	0.51	0.55	0.54	0.52	0.60	0.59	0.57	0.55	0.57
	**5VA1-IFD-5**	0.56	0.59	0.61	0.61	0.61	0.62	0.67	0.70	0.72	0.57	0.63	0.61	0.62	0.67	0.68	0.66	0.65	0.63
**MD conformations**	**5VA1-MD-a**	0.51	0.55	0.55	0.55	0.55	0.60	0.63	0.70	0.71	0.49	0.50	0.49	0.48	0.55	0.56	0.60	0.61	0.62
	**5VA1-MD-b**	0.55	0.58	0.58	0.59	0.56	0.57	0.62	0.69	0.70	0.50	0.52	0.52	0.51	0.60	0.62	0.61	0.65	0.68
	**7CN1**	0.41	0.49	0.49	0.51	0.51	0.52	0.58	0.60	0.59	0.42	0.46	0.43	0.40	0.51	0.54	0.48	0.50	0.49
**DSs/IFs based**																			
**Starting Structures**	**5VA1**	0.56	0.63	0.63	0.65	0.64	0.66	0.69	0.73	0.74	0.48	0.55	0.57	0.59	0.63	0.62	0.65	0.67	0.68
	**MthK-Homo**	0.42	0.51	0.53	0.55	0.59	0.61	0.61	0.68	0.68	0.59	0.65	0.63	0.65	0.69	0.69	0.70	0.72	0.71
	**KvAP -Homo**	0.54	0.59	0.59	0.60	0.64	0.66	0.67	0.74	0.74	0.58	0.62	0.64	0.66	0.69	0.70	0.73	0.74	0.74
**IFD conformations**	**5VA1-IFD-1**	0.56	0.61	0.64	0.63	0.65	0.67	0.72	0.76	0.76	0.58	0.62	0.64	0.65	0.69	0.71	0.72	0.76	0.76
	**5VA1-IFD-2**	0.60	0.63	0.64	0.66	0.70	0.70	0.73	0.75	0.76	0.60	0.65	0.66	0.68	0.70	0.72	0.72	0.76	0.75
	**5VA1-MD-3**	0.59	0.63	0.66	0.68	0.68	0.70	0.73	0.77	0.77	0.60	0.63	0.66	0.67	0.71	0.74	0.73	0.75	0.76
	**5VA1-MD-4**	0.58	0.62	0.65	0.65	0.64	0.66	0.69	0.70	0.72	0.54	0.58	0.58	0.58	0.64	0.64	0.63	0.66	0.68
	**5VA1-IFD-5**	0.58	0.63	0.65	0.66	0.67	0.67	0.72	0.77	0.78	0.55	0.62	0.60	0.63	0.68	0.69	0.69	0.72	0.71
**MD conformations**	**5VA1-IFD-a**	0.54	0.60	0.63	0.64	0.67	0.69	0.72	0.74	0.75	0.59	0.64	0.66	0.68	0.71	0.71	0.73	0.74	0.74
	**5VA1-IFD-b**	0.53	0.60	0.61	0.62	0.64	0.65	0.67	0.73	0.74	0.54	0.60	0.60	0.61	0.63	0.63	0.63	0.69	0.72
	**7CN1**	0.53	0.59	0.60	0.61	0.63	0.63	0.68	0.72	0.71	0.56	0.61	0.61	0.60	0.64	0.64	0.64	0.67	0.66

Table S5

***hERG* conformation**	***BS Volume (Å^3)***
5VA1	587.90
MthK-Homo	159.49
KvAP -Homo	431.15
5VA1-IFD-1	789.56
5VA1-IFD-2	726.18
5VA1-IFD-3	700.34
5VA1-IFD-4	564.85
5VA1-IFD-5	601.62
5VA1-MD-a	685.85
5VA1-MD-b	673.31
7CN1	667.16

Table S6

5VA1	MthK-Homo	KvAP -Homo	5VA1-IFD-1	5VA1-IFD-2	5VA1-IFD-3	5VA1-IFD-4	5VA1-IFD-5	5VA1-MD-a	5VA1-MD-b
657_backbone[99]	653_backbone [97]	656_aromatic[62]	557_aromatic[100]	554_contact[100]	557_aromatic[100]	656_aromatic[100]	621_backbone[100]	623_polar[100]	656_aromatic[100]
657_contact[99]	656_aromatic [97]	656_contact[62]	557_contact[100]	557_backbone[100]	557_contact[100]	656_contact[100]	621_contact[100]	623_sidechain[100]	656_contact[100]
660_contact[92]	656_contact [97]	656_hydrophobic[62]	557_hydrophobic[100]	558_backbone[100]	557_hydrophobic[100]	656_hydrophobic[100]	625_contact[100]	651_contact[100]	656_hydrophobic[100]
660_polar[92]	656_hydrophobic[97]	656_sidechain [62]	557_sidechain[100]	558_contact[100]	557_sidechain[100]	656_sidechain[100]	625_hydrophobic[100]	651_hydrophobic[100]	656_sidechain[100]
660_sidechain[92]	656_sidechain [97]		649_backbone[100]	619_aromatic[100]	622_hydrophobic[100]	653_backbone[95]	625_sidechain[100]	651_sidechain[100]	645_contact[66]
656_backbone[15]	653_contact[88]		655_contact[100]	619_contact[100]	622_sidechain[100]	656_backbone[93]	645_backbone[100]	619_aromatic[97]	645_backbone[65]
653_backbone[1]	654_backbone[43]		655_hydrophobic[100]	619_hydrophobic[100]	623_backbone[100]	653_hydrophobic[88]	645_contact[100]	619_contact[97]	624_contact[31]
	654_contact[36]		655_sidechain[100]	619_sidechain[100]	623_contact[100]	653_sidechain[88]	653_contact[100]	619_hydrophobic[97]	624_polar[31]
	654_polar[18]		656_backbone[100]	622_contact[100]	649_backbone[100]	653_contact[57]	656_contact[100]	619_sidechain[97]	624_sidechain[31]
	654_sidechain[18]		649_contact[98]	622_hydrophobic[100]	649_contact[100]	657_backbone[45]	649_backbone[99]	622_contact[97]	623_contact[9]
	653_hydrophobic[9]		651_hydrophobic[98]	622_sidechain[100]	651_contact[100]	657_contact[45]	652_backbone[99]	557_aromatic[96]	621_backbone[3]
	653_sidechain[9]		651_sidechain[98]	646_contact[100]	651_hydrophobic[100]	660_contact[20]	653_backbone[99]	557_contact[96]	621_contact[3]
			652_backbone[93]	646_hydrophobic[100]	651_sidechain[100]	660_polar[20]	649_contact[98]	557_hydrophobic[96]	652_backbone[1]
			656_contact[91]	646_sidechain[100]	649_polar[99]	660_sidechain[20]	623_polar[97]	557_sidechain[96]	
			651_backbone[89]	649_backbone[100]	649_sidechain[99]	652_backbone[3]	623_sidechain[97]	622_hydrophobic[94]	
			656_aromatic[89]	649_contact[100]	656_contact[99]		656_backbone[97]	622_sidechain[94]	
			656_hydrophobic[89]	649_polar[100]	656_aromatic[98]		648_backbone[94]	622_backbone[93]	
			656_sidechain[89]	649_sidechain[100]	656_hydrophobic[98]		648_contact[94]	652_backbone[89]	
			651_contact[89]	650_backbone[100]	656_sidechain[98]		645_hydrophobic[92]	645_contact[71]	
			652_aromatic[32]	650_contact[100]	560_contact[97]		645_sidechain[92]	645_backbone[70]	
			652_hydrophobic[32]	656_aromatic[100]	560_hydrophobic[97]		656_aromatic[92]	651_backbone[68]	
			652_sidechain[32]	656_hydrophobic[100]	560_sidechain[97]		656_hydrophobic[92]	649_polar[62]	
			649_polar[28]	656_sidechain[100]	650_contact[97]		656_sidechain[92]	649_sidechain[62]	
			649_sidechain[28]	554_hydrophobic[99]	554_contact[96]		649_polar[90]	648_backbone[61]	
			653_hydrophobic[25]	554_sidechain[99]	554_hydrophobic[96]		649_sidechain[90]	648_contact[61]	
			653_sidechain[25]	557_aromatic[99]	554_sidechain[96]		622_backbone[86]	623_backbone[57]	
			655_backbone[14]	557_contact[99]	659_contact[96]		622_contact[86]	649_contact[50]	
			653_contact[9]	557_hydrophobic[99]	659_hydrophobic[96]		653_hydrophobic[86]	557_backbone[40]	
			553_backbone[7]	557_sidechain[99]	659_sidechain[96]		653_sidechain[86]	623_contact[38]	
			553_contact[7]	656_contact[99]	650_backbone[94]		660_contact[62]	646_hydrophobic[27]	
			623_backbone[5]	653_hydrophobic[98]	650_hydrophobic[94]		660_polar[62]	646_sidechain[27]	
				653_sidechain[98]	650_sidechain[94]		660_sidechain[62]	646_contact[24]	
				561_contact[96]	622_contact[93]		652_aromatic[41]	624_contact[3]	
				561_hydrophobic[96]	655_hydrophobic[69]		652_hydrophobic[41]	624_polar[3]	
				561_sidechain[96]	655_sidechain[69]		652_sidechain[41]	624_sidechain[3]	
				646_backbone[92]	655_contact[66]		657_backbone[37]		
				650_hydrophobic[89]	622_backbone[59]		657_contact[37]		
				650_sidechain[89]	623_polar[35]		624_polar[33]		
				653_contact[89]	623_sidechain[35]		624_sidechain[33]		
				554_backbone[87]	651_backbone[31]		624_contact[31]		
				623_polar[50]	647_contact[16]		623_contact[29]		
				623_sidechain[50]	647_hydrophobic[16]		623_backbone[23]		
				652_aromatic[34]	647_sidechain[16]				
				652_contact[34]	624_contact[16]				
				652_hydrophobic[34]	624_polar[16]				
				652_sidechain[34]	624_sidechain[16]				
				619_backbone[22]					
				553_backbone[14]					
				553_contact[14]					
				558_hydrophobic[12]					
				558_sidechain[12]					
				649_acceptor[8]					
