# Supplementary material for: Structure-Based Prediction of hERG-Related Cardiotoxicity: A Benchmark Study
Source: J Chem Inf Model. 2021 Sep 10;61(9):4758–70. doi: 10.1021/acs.jcim.1c00744 (PMC9282647; doi:10.1021/acs.jcim.1c00744)
Supplement: Supplementary file 2 — ci1c00744_si_002.pdf [file ci1c00744_si_002.pdf]

Structure-based prediction of hERG-related cardiotoxicity: a benchmark study

Teresa Maria Creanza[a, Ω], *Pietro Delre*[b,c, Ω], *Nicola Ancona*[a], *Giovanni Lentini*[d], *Michele Saviano*[c] *and Giuseppe Felice Mangiatordi*[c *]

a CNR - Institute of Intelligent Industrial Technologies and Systems for Advanced Manufacturing, Via Amendola 122/o, 70126 Bari, Italy

b Chemistry Department, University of Bari "Aldo Moro", via E. Orabona, 4, I-70125 Bari, Italy.

c CNR – Institute of Crystallography, Via Amendola 122/o, 70126 Bari, Italy

d Department of Pharmacy - Pharmaceutical Sciences, University of Bari "Aldo Moro", via E. Orabona, 4, I-70125 Bari, Italy

*Correspondence: giuseppe.mangiatordi@ic.cnr.it (G.F.M.); +39-080-5929158 (G.F.M.);

[Ω]: These authors contributed equally to this study.

Supplementary material - Table of content

Details on data processing.

Figure S1. Top-scored docking poses returned by IFD simulations performed on five representative *hERG* binders: A) CHEMBL271066, B) CHEMBL1257698, C) CHEMBL3775456, D) CHEMBL3422978 and E) CHEMBL2146867.

Table S1. Kolmogorov-Smirnov test P-values summarizing the difference in docking score distributions between *hERG* binders and non-binders.

Table S2. Sensitivity of all the developed classifiers on the basis of docking scores (top) and docking scores and Ifs (bottom) using GLIDE (left) and GOLD (right) as software. Notice that different thresholds (μM) were considered as described in the "materials and methods" section.

Table S3. Specificity computed for all the developed classifiers on the basis of docking scores (top) and docking scores and Ifs (bottom) using GLIDE (left) and GOLD (right) as software. Notice that different thresholds (μM) were considered as described in the "materials and methods" section.

Table S4. Interactions responsible for a lower IC_{50} based on a KS test performed on the IFs returned by all the considered protein models. The table shows the interactions sorted by the number of occurrences of significant KS test p-values ($p < 0.05$) in the 100 trials (the occurence is shown in square brackets).

Details on data processing

All data analysis was completed in MATLAB using the Statistics and Machine Learning Toolbox. In particular, the function kstest2 was used to perform the Kolmogorov-Smirnov test in order to compare two sample distributions. The function fitcsvm was used to train support vector machine model for two-class classification based on docking score values of hERG binders and nonbinders (low-dimensional predictor data set). In order to process docking scores and protein-ligand interaction fingerprints, the function fitclinear was used to train regularized support vector machines for binary learning with high-dimensional and sparse predictor data. Fitclinear minimizes the objective function using stochastic gradient descent to reduce computing time.

Figure S1

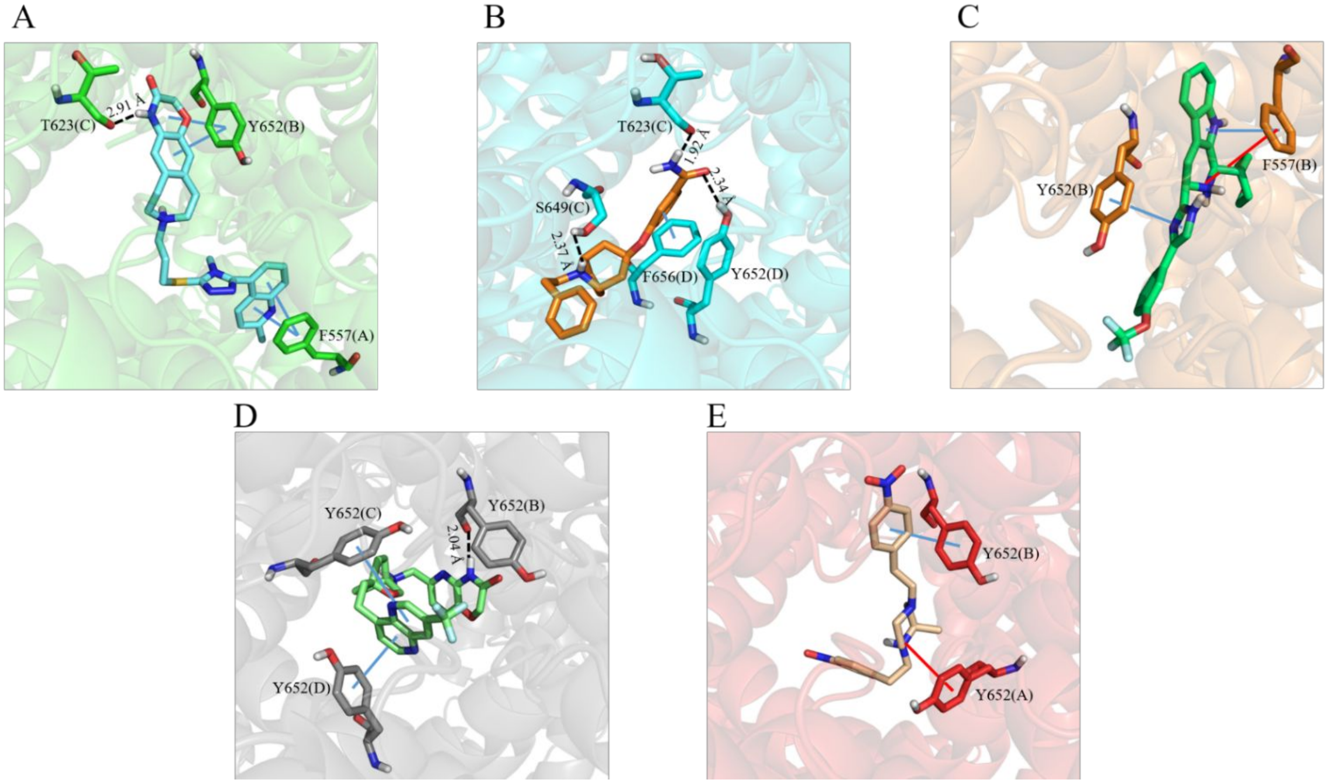

A
T623(C)
2.91 Å
Y652(B)
F557(A)
B
T623(C)
1.92 Å
2.34 Å
S649(C)
2.37 Å
F656(D)
Y652(D)
C
Y652(B)
F557(B)
D
Y652(C)
Y652(B)
2.04 Å
Y652(D)
E
Y652(B)
Y652(A)

Table S1

Threshold (μM)		1	10	20	30	40	50	60	70	80
						GLIDE				
Starting Structures	5VA1	6.00E-49	7.46E-71	4.31E-67	6.31E-71	2.48E-62	9.62E-65	1.26E-74	3.11E-75	2.84E-76
	MthK-Homo	1.54E-20	7.08E-34	1.71E-31	6.11E-34	5.90E-27	3.57E-27	1.31E-29	2.91E-30	5.53E-29
	KvAP -Homo	6.17E-27	1.00E-38	2.19E-38	6.92E-41	4.77E-41	4.84E-43	1.15E-47	9.54E-53	1.55E-51
IFD conformations	5VA1-IFD-1	7.45E-59	1.81E-82	2.00E-89	3.35E-94	3.75E-100	1.69E-105	3.45E-121	5.05E-139	5.64E-138
	5VA1-IFD-2	2.99E-47	2.61E-73	4.70E-83	5.75E-90	2.26E-94	7.54E-98	4.17E-112	1.14E-129	7.31E-129
	5VA1-IFD-3	1.94E-48	6.09E-71	1.98E-78	6.96E-84	1.60E-79	5.77E-84	9.92E-98	7.61E-118	7.99E-119
	5VA1-IFD-4	5.57E-45	6.17E-68	7.86E-73	7.24E-77	9.56E-69	4.19E-73	8.84E-84	3.30E-90	2.07E-89
	5VA1-IFD-5	6.39E-60	1.22E-85	5.69E-89	1.37E-96	6.54E-88	1.04E-91	1.60E-101	1.76E-113	1.28E-118
MD conformations	5VA1-MD-a	2.31E-43	4.62E-63	3.86E-73	8.75E-81	5.38E-94	8.70E-103	3.40E-117	5.03E-136	2.36E-135
	5VA1-MD-b	4.94E-40	1.02E-59	3.59E-65	3.94E-72	1.27E-78	3.71E-84	1.44E-98	8.90E-116	3.95E-116
	7CN1	3.57E-35	1.71E-56	9.97E-57	5.82E-62	1.07E-64	2.81E-66	2.81E-71	4.23E-80	2.51E-77
						GOLD				
Starting Structure	5VA1	1.67E-24	5.78E-33	3.55E-26	2.29E-24	3.11E-27	1.33E-27	4.57E-25	1.70E-20	1.15E-18
	MthK-Homo	9.45E-65	4.65E-90	3.57E-90	3.20E-88	1.61E-99	5.41E-100	1.30E-102	1.25E-99	3.43E-96
	KvAP -Homo	8.30E-41	1.14E-44	2.55E-46	6.65E-43	1.18E-66	6.01E-67	1.15E-70	7.75E-83	3.15E-81
IFD conformations	5VA1-IFD-1	5.81E-44	6.55E-67	4.98E-77	1.57E-78	2.77E-105	5.30E-109	5.93E-111	1.21E-122	8.27E-122
	5VA1-IFD-2	1.00E-67	1.69E-90	4.52E-97	6.40E-100	3.49E-128	1.97E-134	1.13E-127	8.71E-131	2.68E-131
	5VA1-IFD-3	1.47E-56	1.09E-78	1.30E-82	7.47E-81	2.04E-104	3.17E-111	1.55E-109	5.43E-117	2.10E-115
	5VA1-IFD-4	2.22E-41	2.22E-56	5.84E-48	1.83E-45	1.62E-55	8.98E-56	5.71E-57	6.71E-56	4.63E-54
	5VA1-IFD-5	5.43E-45	2.32E-62	2.41E-59	2.44E-57	5.44E-73	2.84E-73	1.97E-65	1.17E-64	8.26E-62
MD conformations	5VA1-MD-a	2.39E-47	3.08E-63	9.08E-72	4.99E-73	2.56E-96	5.48E-99	2.42E-104	1.31E-113	5.93E-113
	5VA1-MD-b	5.03E-32	4.53E-45	2.08E-45	3.00E-43	1.94E-65	1.24E-67	1.54E-68	1.94E-75	4.63E-76
	7CN1	6.53E-18	9.67E-24	9.67E-24	2.01E-20	1.68E-30	3.00E-30	3.68E-29	9.00E-30	6.59E-28

Table S2

Software		GLIDE									GOLD								
**Threshold (μM)**		**1**	**10**	**20**	**30**	**40**	**50**	**60**	**70**	**80**	**1**	**10**	**20**	**30**	**40**	**50**	**60**	**70**	**80**
**DSs based**																			
**Starting Structures**	**5VA1**	0.70	0.69	0.67	0.68	0.71	0.72	0.70	0.70	0.71	0.68	0.70	0.74	0.77	0.72	0.74	0.77	0.76	0.74
	**MthK-Homo**	0.66	0.63	0.63	0.61	0.56	0.58	0.57	0.59	0.57	0.68	0.70	0.71	0.72	0.73	0.72	0.74	0.76	0.76
	**KvAP -Homo**	0.58	0.60	0.60	0.60	0.60	0.59	0.61	0.62	0.59	0.71	0.72	0.74	0.75	0.75	0.78	0.78	0.78	0.79
**IFD conformations**	**5VA1-IFD-1**	0.71	0.73	0.74	0.75	0.78	0.80	0.79	0.80	0.79	0.70	0.72	0.74	0.74	0.79	0.79	0.81	0.81	0.82
	**5VA1-IFD-2**	0.69	0.69	0.71	0.72	0.76	0.76	0.75	0.76	0.77	0.75	0.76	0.79	0.81	0.81	0.81	0.82	0.83	0.82
	**5VA1-IFD-3**	0.65	0.64	0.66	0.65	0.71	0.73	0.77	0.75	0.76	0.74	0.73	0.75	0.78	0.78	0.78	0.80	0.81	0.81
	**5VA1-IFD-4**	0.71	0.71	0.74	0.74	0.76	0.77	0.74	0.75	0.76	0.68	0.68	0.68	0.71	0.70	0.72	0.74	0.77	0.76
	**5VA1-IFD-5**	0.70	0.71	0.74	0.74	0.77	0.76	0.77	0.78	0.80	0.63	0.63	0.65	0.63	0.67	0.68	0.69	0.71	0.71
**MD conformations**	**5VA1-MD-a**	0.70	0.71	0.75	0.76	0.83	0.83	0.83	0.83	0.84	0.71	0.75	0.78	0.80	0.84	0.85	0.85	0.87	0.86
	**5VA1-MD-b**	0.66	0.68	0.69	0.70	0.80	0.79	0.81	0.79	0.80	0.67	0.69	0.72	0.73	0.73	0.71	0.72	0.74	0.73
	**7CN1**	0.77	0.75	0.77	0.78	0.80	0.81	0.80	0.81	0.80	0.71	0.68	0.73	0.75	0.71	0.69	0.73	0.74	0.73
**DSs/IFs based**																			
**Starting Structure**	**5VA1**	0.68	0.68	0.70	0.72	0.75	0.76	0.76	0.78	0.78	0.73	0.72	0.73	0.71	0.76	0.77	0.77	0.77	0.77
	**MthK-Homo**	0.70	0.67	0.68	0.67	0.67	0.66	0.66	0.66	0.65	0.67	0.68	0.70	0.72	0.73	0.75	0.77	0.78	0.79
	**KvAP -Homo**	0.64	0.65	0.67	0.67	0.69	0.68	0.71	0.72	0.71	0.64	0.65	0.67	0.68	0.69	0.71	0.73	0.75	0.77
**IFD conformations**	**5VA1-IFD-1**	0.67	0.71	0.72	0.73	0.77	0.78	0.79	0.81	0.81	0.64	0.65	0.69	0.69	0.76	0.76	0.76	0.78	0.79
	**5VA1-IFD-2**	0.61	0.65	0.68	0.69	0.73	0.74	0.76	0.77	0.79	0.68	0.70	0.73	0.74	0.78	0.78	0.80	0.80	0.80
	**5VA1-IFD-3**	0.65	0.66	0.68	0.68	0.71	0.71	0.75	0.76	0.77	0.63	0.63	0.67	0.71	0.74	0.74	0.77	0.78	0.78
	**5VA1-IFD-4**	0.67	0.66	0.70	0.70	0.73	0.75	0.74	0.76	0.77	0.67	0.68	0.68	0.70	0.71	0.73	0.75	0.75	0.75
	**5VA1-IFD-5**	0.68	0.68	0.72	0.73	0.77	0.77	0.79	0.80	0.81	0.67	0.67	0.68	0.70	0.72	0.72	0.74	0.76	0.77
**MD conformations**	**5VA1-MD-a**	0.70	0.70	0.70	0.70	0.74	0.77	0.79	0.81	0.82	0.66	0.65	0.70	0.70	0.72	0.74	0.75	0.80	0.78
	**5VA1-MD-b**	0.69	0.69	0.70	0.71	0.77	0.76	0.79	0.79	0.80	0.65	0.67	0.71	0.73	0.76	0.75	0.76	0.78	0.77
	**7CN1**	0.68	0.67	0.71	0.72	0.75	0.77	0.79	0.81	0.81	0.66	0.65	0.67	0.66	0.69	0.69	0.71	0.73	0.74

Table S3

Software		GLIDE									GOLD								
**Threshold (μM)**		**1**	**10**	**20**	**30**	**40**	**50**	**60**	**70**	**80**	**1**	**10**	**20**	**30**	**40**	**50**	**60**	**70**	**80**
**DSs based**																			
**Starting Structures**	**5VA1**	0.52	0.60	0.61	0.62	0.63	0.62	0.67	0.70	0.70	0.45	0.45	0.43	0.41	0.46	0.48	0.42	0.42	0.43
	**MthK-Homo**	0.48	0.55	0.56	0.59	0.64	0.64	0.65	0.65	0.67	0.58	0.62	0.61	0.63	0.67	0.67	0.70	0.68	0.70
	**KvAP -Homo**	0.56	0.61	0.61	0.62	0.64	0.65	0.68	0.72	0.74	0.50	0.49	0.47	0.47	0.56	0.57	0.58	0.63	0.63
**IFD conformations**	**5VA1-IFD-1**	0.54	0.57	0.59	0.59	0.61	0.63	0.67	0.74	0.74	0.50	0.55	0.57	0.59	0.63	0.63	0.64	0.69	0.68
	**5VA1-IFD-2**	0.54	0.58	0.61	0.62	0.64	0.65	0.69	0.75	0.75	0.51	0.55	0.55	0.55	0.65	0.68	0.66	0.69	0.70
	**5VA1-IFD-3**	0.57	0.63	0.65	0.67	0.63	0.64	0.67	0.72	0.73	0.49	0.53	0.57	0.55	0.63	0.67	0.64	0.67	0.68
	**5VA1-IFD-4**	0.49	0.54	0.58	0.57	0.57	0.58	0.63	0.67	0.68	0.51	0.55	0.54	0.52	0.60	0.59	0.57	0.55	0.57
	**5VA1-IFD-5**	0.56	0.59	0.61	0.61	0.61	0.62	0.67	0.70	0.72	0.57	0.63	0.61	0.62	0.67	0.68	0.66	0.65	0.63
**MD conformations**	**5VA1-MD-a**	0.51	0.55	0.55	0.55	0.55	0.60	0.63	0.70	0.71	0.49	0.50	0.49	0.48	0.55	0.56	0.60	0.61	0.62
	**5VA1-MD-b**	0.55	0.58	0.58	0.59	0.56	0.57	0.62	0.69	0.70	0.50	0.52	0.52	0.51	0.60	0.62	0.61	0.65	0.68
	**7CN1**	0.41	0.49	0.49	0.51	0.51	0.52	0.58	0.60	0.59	0.42	0.46	0.43	0.40	0.51	0.54	0.48	0.50	0.49
**DSs/IFs based**																			
**Starting Structures**	**5VA1**	0.56	0.63	0.63	0.65	0.64	0.66	0.69	0.73	0.74	0.48	0.55	0.57	0.59	0.63	0.62	0.65	0.67	0.68
	**MthK-Homo**	0.42	0.51	0.53	0.55	0.59	0.61	0.61	0.68	0.68	0.59	0.65	0.63	0.65	0.69	0.69	0.70	0.72	0.71
	**KvAP -Homo**	0.54	0.59	0.59	0.60	0.64	0.66	0.67	0.74	0.74	0.58	0.62	0.64	0.66	0.69	0.70	0.73	0.74	0.74
**IFD conformations**	**5VA1-IFD-4**	0.56	0.61	0.64	0.63	0.65	0.67	0.72	0.76	0.76	0.58	0.62	0.64	0.65	0.69	0.71	0.72	0.76	0.76
	**5VA1-IFD-5**	0.60	0.63	0.64	0.66	0.70	0.70	0.73	0.75	0.76	0.60	0.65	0.66	0.68	0.70	0.72	0.72	0.76	0.75
	**5VA1-MD-a**	0.59	0.63	0.66	0.68	0.68	0.70	0.73	0.77	0.77	0.60	0.63	0.66	0.67	0.71	0.74	0.73	0.75	0.76
	**5VA1-MD-b**	0.58	0.62	0.65	0.65	0.64	0.66	0.69	0.70	0.72	0.54	0.58	0.58	0.58	0.64	0.64	0.63	0.66	0.68
	**5VA1-IFD-1**	0.58	0.63	0.65	0.66	0.67	0.67	0.72	0.77	0.78	0.55	0.62	0.60	0.63	0.68	0.69	0.69	0.72	0.71
**MD conformations**	**5VA1-IFD-2**	0.54	0.60	0.63	0.64	0.67	0.69	0.72	0.74	0.75	0.59	0.64	0.66	0.68	0.71	0.71	0.73	0.74	0.74
	**5VA1-IFD-3**	0.53	0.60	0.61	0.62	0.64	0.65	0.67	0.73	0.74	0.54	0.60	0.60	0.61	0.63	0.63	0.63	0.69	0.72
	**7CN1**	0.53	0.59	0.60	0.61	0.63	0.63	0.68	0.72	0.71	0.56	0.61	0.61	0.60	0.64	0.64	0.64	0.67	0.66

Table S4.

5VA1	MthK-Homo	KvAP -Homo	5VA1-IFD-1	5VA1-IFD-2	5VA1-IFD-3	5VA1-IFD-4	5VA1-IFD-5	5VA1-MD-a	5VA1-MD-b
657_backbone[99]	653_backbone [97]	656_aromatic[62]	557_aromatic[100]	554_contact[100]	557_aromatic[100]	656_aromatic[100]	621_backbone[100]	623_polar[100]	656_aromatic[100]
657_contact[99]	656_aromatic [97]	656_contact[62]	557_contact[100]	557_backbone[100]	557_contact[100]	656_contact[100]	621_contact[100]	623_sidechain[100]	656_contact[100]
660_contact[92]	656_contact [97]	656_hydrophobic[62]	557_hydrophobic[100]	558_backbone[100]	557_hydrophobic[100]	656_hydrophobic[100]	625_contact[100]	651_contact[100]	656_hydrophobic[100]
660_polar[92]	656_hydrophobic[97]	656_sidechain [62]	557_sidechain[100]	558_contact[100]	557_sidechain[100]	656_sidechain[100]	625_hydrophobic[100]	651_hydrophobic[100]	656_sidechain[100]
660_sidechain[92]	656_sidechain [97]		649_backbone[100]	619_aromatic[100]	622_hydrophobic[100]	653_backbone[95]	625_sidechain[100]	651_sidechain[100]	645_contact[66]
656_backbone[15]	653_contact[88]		655_contact[100]	619_contact[100]	622_sidechain[100]	656_backbone[93]	645_backbone[100]	619_aromatic[97]	645_backbone[65]
653_backbone[1]	654_backbone[43]		655_hydrophobic[100]	619_hydrophobic[100]	623_backbone[100]	653_hydrophobic[88]	645_contact[100]	619_contact[97]	624_contact[31]
	654_contact[36]		655_sidechain[100]	619_sidechain[100]	623_contact[100]	653_sidechain[88]	653_contact[100]	619_hydrophobic[97]	624_polar[31]
	654_polar[18]		656_backbone[100]	622_contact[100]	649_backbone[100]	653_contact[57]	656_contact[100]	619_sidechain[97]	624_sidechain[31]
	654_sidechain[18]		649_contact[98]	622_hydrophobic[100]	649_contact[100]	657_backbone[45]	649_backbone[99]	622_contact[97]	623_contact[9]
	653_hydrophobic[9]		651_hydrophobic[98]	622_sidechain[100]	651_contact[100]	657_contact[45]	652_backbone[99]	557_aromatic[96]	621_backbone[3]
	653_sidechain[9]		651_sidechain[98]	646_contact[100]	651_hydrophobic[100]	660_contact[20]	653_backbone[99]	557_contact[96]	621_contact[3]
			652_backbone[93]	646_hydrophobic[100]	651_sidechain[100]	660_polar[20]	649_contact[98]	557_hydrophobic[96]	652_backbone[1]
			656_contact[91]	646_sidechain[100]	649_polar[99]	660_sidechain[20]	623_polar[97]	557_sidechain[96]	
			651_backbone[89]	649_backbone[100]	649_sidechain[99]	652_backbone[3]	623_sidechain[97]	622_hydrophobic[94]	
			656_aromatic[89]	649_contact[100]	656_contact[99]		656_backbone[97]	622_sidechain[94]	
			656_hydrophobic[89]	649_polar[100]	656_aromatic[98]		648_backbone[94]	622_backbone[93]	
			656_sidechain[89]	649_sidechain[100]	656_hydrophobic[98]		648_contact[94]	652_backbone[89]	
			651_contact[89]	650_backbone[100]	656_sidechain[98]		645_hydrophobic[92]	645_contact[71]	
			652_aromatic[32]	650_contact[100]	560_contact[97]		645_sidechain[92]	645_backbone[70]	
			652_hydrophobic[32]	656_aromatic[100]	560_hydrophobic[97]		656_aromatic[92]	651_backbone[68]	
			652_sidechain[32]	656_hydrophobic[100]	560_sidechain[97]		656_hydrophobic[92]	649_polar[62]	
			649_polar[28]	656_sidechain[100]	650_contact[97]		656_sidechain[92]	649_sidechain[62]	
			649_sidechain[28]	554_hydrophobic[99]	554_contact[96]		649_polar[90]	648_backbone[61]	
			653_hydrophobic[25]	554_sidechain[99]	554_hydrophobic[96]		649_sidechain[90]	648_contact[61]	
			653_sidechain[25]	557_aromatic[99]	554_sidechain[96]		622_backbone[86]	623_backbone[57]	
			655_backbone[14]	557_contact[99]	659_contact[96]		622_contact[86]	649_contact[50]	
			653_contact[9]	557_hydrophobic[99]	659_hydrophobic[96]		653_hydrophobic[86]	557_backbone[40]	
			553_backbone[7]	557_sidechain[99]	659_sidechain[96]		653_sidechain[86]	623_contact[38]	
			553_contact[7]	656_contact[99]	650_backbone[94]		660_contact[62]	646_hydrophobic[27]	
			623_backbone[5]	653_hydrophobic[98]	650_hydrophobic[94]		660_polar[62]	646_sidechain[27]	
				653_sidechain[98]	650_sidechain[94]		660_sidechain[62]	646_contact[24]	
				561_contact[96]	622_contact[93]		652_aromatic[41]	624_contact[3]	
				561_hydrophobic[96]	655_hydrophobic[69]		652_hydrophobic[41]	624_polar[3]	
				561_sidechain[96]	655_sidechain[69]		652_sidechain[41]	624_sidechain[3]	
				646_backbone[92]	655_contact[66]		657_backbone[37]		
				650_hydrophobic[89]	622_backbone[59]		657_contact[37]		
				650_sidechain[89]	623_polar[35]		624_polar[33]		
				653_contact[89]	623_sidechain[35]		624_sidechain[33]		
				554_backbone[87]	651_backbone[31]		624_contact[31]		
				623_polar[50]	647_contact[16]		623_contact[29]		
				623_sidechain[50]	647_hydrophobic[16]		623_backbone[23]		
				652_aromatic[34]	647_sidechain[16]				
				652_contact[34]	624_contact[16]				
				652_hydrophobic[34]	624_polar[16]				
				652_sidechain[34]	624_sidechain[16]				
				619_backbone[22]					
				553_backbone[14]					
				553_contact[14]					
				558_hydrophobic[12]					
				558_sidechain[12]					
				649_acceptor[8]					
